# Neuroprotective Effects of Phytochemicals against Aluminum Chloride-Induced Alzheimer’s Disease through ApoE4/LRP1, Wnt3/β-Catenin/GSK3β, and TLR4/NLRP3 Pathways with Physical and Mental Activities in a Rat Model

**DOI:** 10.3390/ph15081008

**Published:** 2022-08-17

**Authors:** Ahmed Mohsen Elsaid Hamdan, Fatimah Hussain J. Alharthi, Ahmed Hadi Alanazi, Soad Z. El-Emam, Sameh S. Zaghlool, Kamel Metwally, Sana Abdulaziz Albalawi, Yahia S. Abdu, Reda El-Sayed Mansour, Hoda A. Salem, Zakaria Y. Abd Elmageed, Karema Abu-Elfotuh

**Affiliations:** 1 Department of Pharmacy Practice, Faculty of Pharmacy, University of Tabuk, Tabuk 71491, Saudi Arabia; 2 Graduate Pharmacist, Faculty of Pharmacy, University of Tabuk, Tabuk 71491, Saudi Arabia; 3 Pharmacist in IV Room, King Fahad Specialist Hospital, Dammam 32253, Saudi Arabia; 4 Pharmacology and Toxicology Department, Faculty of Pharmacy, October 6 University, Giza 12585, Egypt; 5 Pharmacology and Toxicology Department, Faculty of Pharmacy, Modern University for Technology and Information (MTI), Mokattam, Cairo 11571, Egypt; 6 Department of Pharmaceutical Chemistry, Faculty of Pharmacy, University of Tabuk, Tabuk 71491, Saudi Arabia; 7 Department of Medicinal Chemistry, Faculty of Pharmacy, Zagazig University, Zagazig 44519, Egypt; 8 Faculty of Medicine, Ain Shams University, Giza 11591, Egypt; 9 Department of Pharmaceutical Medicinal Chemistry and Drug Design, Faculty of Pharmacy (Girls), Al-Azhar University, Cairo 11651, Egypt; 10 Department of Clinical Pharmacy, Faculty of Pharmacy, Al-Azhar University, Cairo 11651, Egypt; 11 Department of Pharmacology, Edward Via College of Osteopathic Medicine, University of Louisiana at Monroe, Monroe, LA 71203, USA; 12 Department of Pharmacology and Toxicology, Faculty of Pharmacy, Al-Azhar University, Cairo 11651, Egypt

**Keywords:** inflammasome, molecular docking, oxidative stress, phytochemicals, amyloid beta, tau, neurodegeneration, NLRP3

## Abstract

Background: Alzheimer’s disease (AD) is a neurodegenerative disorder that is associated with abnormal cognition. AD is aided in its initiation and progression by hereditary and environmental factors. Aluminum (Al) is a neurotoxic agent that causes oxidative stress, which is linked to AD progression. Additionally, Nrf2/HO-1, APOE4/LRP1, Wnt3/β-catenin, and TLR4/NLRP3 are the main signaling pathways involved in AD pathogenesis. Several phytochemicals are promising options in delaying AD evolution. Objectives: This study aimed at studying the neuroprotective effects of some phytochemicals as morin (MOR), thymol (TML), and thymoquinone (TMQ) on physical and mental activities (PhM) in Al chloride (AlCl_3_)-induced AD rat model. Another objective was to determine the specificity of phytochemicals to AD signaling pathways using molecular docking. Methods: Eighty male Dawley rats were divided into eight groups. Each group received: saline (control group), AlCl_3_, (ALAD), PhM, either alone or with a combination of MOR, TML, and/or TMQ for five weeks. Animals were then subjected to behavioral evaluation. Brain tissues were used for histopathological and biochemical analyses to determine the extent of neurodegeneration. The effect of phytochemicals on AlCl_3_-induced oxidative stress and the main signaling pathways involved in AD progression were also investigated. Results: AlCl_3_ caused a decline in spatial learning and memory, as well as histopathological changes in the brains of rats. Phytochemicals combined with PhM restored antioxidant activities, increased HO-1 and Nrf2 levels, blocked inflammasome activation, apoptosis, TLR4 expression, amyloide-β generation, and tau hyperphophorylation. They also brought ApoE4 and LRP1 levels back to normal and regulated Wnt3/β-catenin/GSK3β signaling pathway. Conclusions: The use of phytochemicals with PhM is a promising strategy for reducing AD by modulating Nrf2/HO-1, TLR4/NLRP3, APOE4/LRP1, and Wnt3/β-catenin/GSK-3β signaling pathways.

## 1. Introduction

Alzheimer’s disease (AD) is an age-related, irreversible, and progressive neurodegenerative disorder characterized by memory loss, cognitive dysfunction, and other behavioral disabilities that interfere with daily activities [1]. These symptoms are due to the irreversible loss of neurons and the formation of abnormal extracellular protein aggregates called amyloid beta (Aβ), which is a major neuropathological feature [2]. Intracellular neurofibrillary tangles (NFTs) of hyperphosphorylated Tau protein, senile plaques (SPs) and synapse loss are among the pathological features of Alzheimer’s disease [3,4,5]. 

Aluminum (Al) contributes to the etiology of several neurodegenerative disorders by affecting several biomolecules related to neurotoxicity [6,7]. Al is known to speed up oxidative stress, the cross-linking and deposition of Aβ oligomers, and the formation of plaques in the brain cortex and hippocampus. Thus, aluminum chloride (AlCl_3_)-induced AD in rats is a good model for studying the neuroprotective effects of many phytochemicals and chemical compounds against AD [8,9,10]. The predominance of Al in the environment, normal life activities and food makes it almost unavoidable for exposure [11,12]. By using this model, it mimics the protective and ameliorating effects of some chemicals against Alzheimer’s disease in humans. 

Aβ production is initiated by the cleavage of amyloid precursor protein (APP) by β-secretase, which is commonly known as beta-site amyloid precursor protein cleaving enzyme 1 (BACE1), and γ-secretase enzymes [13]. Aβ can diffuse readily through the brain parenchyma and activate a cascade of pathogenic events such as neuronal apoptosis/necrosis, induction of oxidative stress and neuroinflammation in the cortex and hippocampus [2,14]. In addition, necrotic brain cells release damage-associated molecular patterns (DAMPs) that activate toll-like receptor 4 (TLR4) in response to such endogenous danger molecules. TLR4 activation initiates the immune response through TLR4/NF-κB cues [15,16]. Aβ binds to TLR4 and induces an inflammatory cascade culminating in activation of the NLRP3 inflammasome multiprotein complex [17], which leads to neurodegeneration. As a result, squelching the TLR4/NLRP3 inflammasome pathway has a significant role in preventing neuroinflammatory-dependent diseases [15,18,19]. It is worth mentioning that the decreased clearance of Aβ leads to its accumulation in the brain and plays a significant role in the pathogenesis of AD [20,21]. The low-density lipoprotein receptor-related protein-1 (LRP1) mediates tau uptake and extracellular Aβ clearance from the brain by cellular degradation or transcytosis across the blood-brain barrier (BBB) [22]. However, these are inhibited by increased levels of apolipoprotein E4 (ApoE4) due to its greater affinity for LRP1 [23]. 

Recently, the Wnt signaling pathway has also been linked to a multitude of neuronal impairments [24]. It has critical role for the self-renewal, fate commitment, and survival of the adult CNS [25]. Wnt/β-catenin signaling has been shown to be involved in cell lineage decisions during developmental stages and in adult brains. The development of therapeutic approaches to inhibit GSK3β and/or induce Wnt/β-catenin signaling in neural cells can mitigate the toxic effects of Aβ and promote neurogenesis in AD patients [1,2] This is because overactivation of GSK3β accounts for several features of this pathology, such as memory impairment, tau phosphorylation, increased amyloid production, microglia-mediated inflammation, and neuronal death [24]. 

Since multiple and interdependent mechanisms are involved in the pathological process of AD, and all end with enhanced oxidative stress and inflammation, we studied the effects of some phytochemicals in addition to physical and mental activities (PhM) as a therapeutic approach for suppressing the progression of AD. Such commonly used phytochemicals are promising oxidative stress and neuroinflammation inhibitors [26,27]. They have been also proven to treat mitochondrial dysfunction [28]. Moreover, phytochemicals can exert neuroprotective effects via scavenging oxygen free radicals directly [29]. They can also modulate the nuclear factor erythroid 2-related factor 2/hemoxygenase-1 (Nrf2/HO-1) pathway to enhance the cellular antioxidant defense [30].

Thymol (THL), a monoterpene phenol isolated from medicinal herbs, has exhibited robust neuroprotective effects. It also exhibits a variety of biological activities, including anti-inflammatory and antioxidant effects [31], and enhances cognitive activities in a model of dementia [32,33], but the exact mechanism has not been fully studied. Thymoquinone (TMQ), the active compound of N. sativa, can prevent learning dysfunction and improve initial latency and step-through latency. It also reduces plaque formation in the hippocampus while increasing the number of neurons and protecting pyramidal cells from the neurotoxic effects of Aβ [34]. Morin (MOR) is a natural polyphenol, originally isolated from members of the Moraceae family. It is found in several fruits and vegetables [35,36] and exerts antioxidant, anti-inflammatory, antitumoral, antibacterial, and neuroprotective effects [37]. 

The aim of this study was to investigate the neuroprotective effect of phytochemicals with PhM against AlCl_3_-induced Alzheimer’s disease using a rat model. This study explored the potential therapeutic and preventive approaches that can be used to minimize the destructive effects of Al to the brain by investigating their impact on ApoE4/LRP1, Wnt3/β-catenin/GSK3β, and TLR4/NLRP3 trajectories. 

## 2. Results

### 2.1. A Combination of MOR, TML, and TMQ with PhM Ameliorates Learning and Memory Impairment in ALAD Rat Model

**Figure 1:**
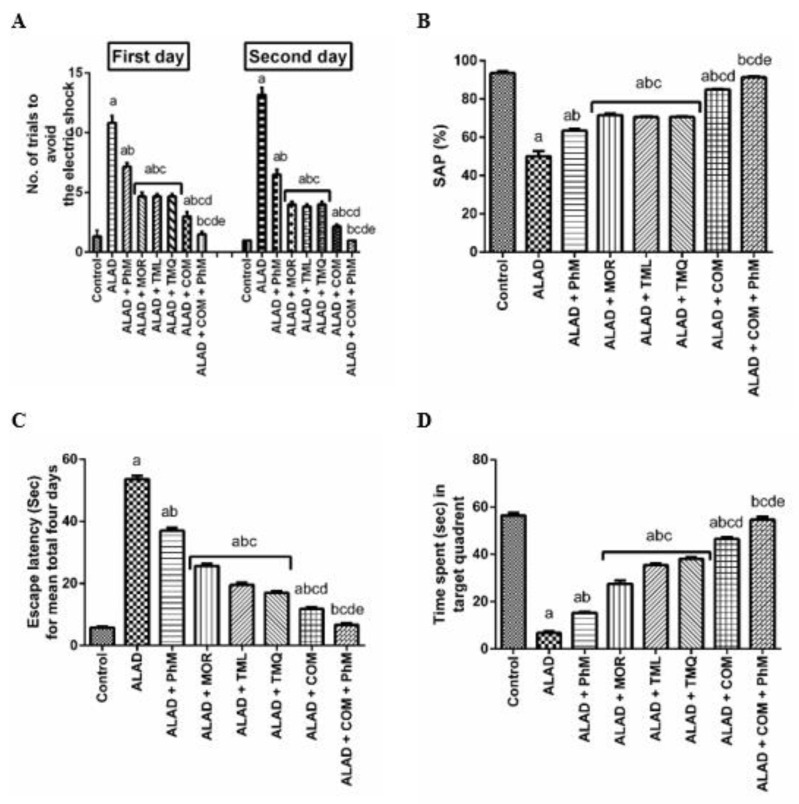
The effect of phytochemical combinations, MOR, TML, and TMQ, with PhM on the behavioral changes induced by AlCl_3_ administration for five weeks (70 mg/kg/day, i.p.). (**A**) The number of trials to avoid the electric shock in CAR test. (**B**) SAP (%) in Y-Maze test. (**C**) The escape latency in four days. (**D**) The time spent in target the quadrant in the MWM test. The data are presented as means ± SD (*n* = 10). Significance (a): relative to the control group. Significance (b): relative to the ALAD group. Significance (c): relative to ALAD + PhM group. Significance (d): relative to either ALAD + MOR, ALAD + TML, or ALAD + TMQ group. Significance (e): relative to ALAD + COM group. Significance: *p* < 0.05. The data of the effect of MOR, TML and TMQ on the control are not shown as they are not significant.

Behavioral studies were used to evaluate the protective effects of phytochemicals combination with PhM against AlCl_3_-induced learning and memory impairment. As shown in Figure 1A, the ALAD group showed an 11-fold elevation in the number of avoidance response to the electric shock compared to the control group. Meanwhile, physical, and mental activity decreased the elevated number of trials for avoidance response to electric shock in the first and second days by about 36% and 50%, respectively, compared to the ALAD group. There were no significant differences among the reduced number of trials of avoidance response to the electric shock in the first and second days for MOR-treated, TML-treated, or TMQ-treated groups alone. These three groups showed a reduction in the number of avoidance response to the electric shock in the first and second days of about 59% and 71%, respectively, compared to the ALAD group. Meanwhile, a combination of MOR, TML and TMQ resulted in a significant reduction in the number of trials for avoidance by about 73% compared to the ALAD group. MOR, TML, and TMQ combination with PhM markedly improved the avoidance response by decreasing the number of trials to evade the electric shock on both the first and second days of the experiment compared to the ALAD group (*p* < 0.05). Additionally, the observed reduction of SAP%, as indicated by the Y-Maze test, was significantly improved by phytochemicals combination (Figure 1B). The results of the MWM test showed that learning and spatial memory deficits induced by AlCl_3_ treatment were amended by the combination therapy and PhM, as evidenced by the gradual reduction in the escape latency over the four days (Figure 1C) and the increased time spent in the target quadrant (Figure 1D), respectively, as compared with the ALAD group (*p* < 0.05). It is noteworthy that TMQ showed the highest restoration of the escape latency and the maximum time spent in the target quadrant compared to MOR and TML (Figure 1C,D). The data for the effect of MOR, TML and TMQ on the control is not shown as it is not significant.

### 2.2. Histopathological Evaluation of Brain Tissues

**Figure 2:**
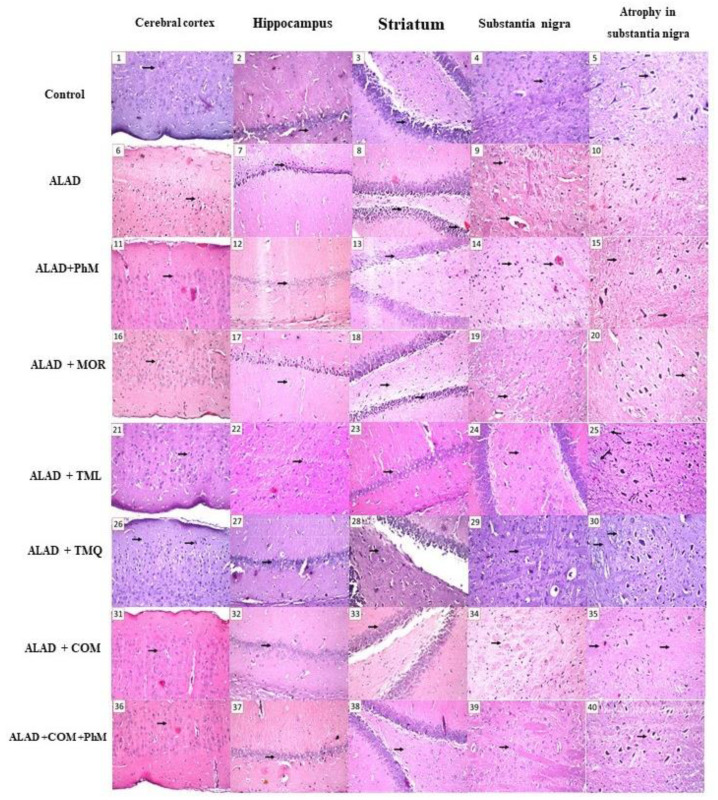
Photomicrographs of brain sections stained by Hematoxylin and Eosin (magnification 40X). In control group, there was no histopathological alteration in the cerebral cortex, hippocampus, striatum, and substantia nigra (**Inserts 1–5**). In ALAD group, there were nuclear pyknosis and degeneration in the neuronal cells of the cerebral cortex (**Insert 6**), subiculum and fascia dentate of the hippocampus (**Inserts 7,8**). Focal eosinophilic plagues were detected in the striatum (**Insert 9**). The substantia nigra showed atrophy in the neuronal cells (**Insert 10**). In ALAD + PhM group, the cerebral cortex and hippocampus showed no histopathological alteration (**Inserts 11–13**). Nuclear pyknosis and degeneration were recorded in the neurons of the striatum with congestion in the blood vessel (**Insert 14**). The substantia nigra showed atrophy in some of the neuronal cells (**Insert 15**). In ALAD + MOR group, there was no histopathological alteration in the cerebral cortex (**Insert 16**). Nuclear pyknosis and degeneration were observed in some neuronal cells of the subiculum as well as the fascia dentate in the hippocampus (**Inserts 17,18**). The striatum showed intracellular oedema in the neuronal cells (**Insert 19**). Mild atrophy was detected in the cells of substantia nigra (**Insert 20**). In ALAD+ TML group, nuclear pyknosis was observed in the neurons of the cerebral cortex and striatum while the hippocampus was intact (**Inserts 21–24**). Diffuse gliosis was detected in substantia nigra **(Insert 25).** In ALAD + TMQ group, the cerebral cortex showed focal nuclear pyknosis and degeneration in the neuronal cells (**Insert 26**). There was no histopathological alteration in the hippocampus as well as in the striatum (**Inserts 27–29**). Atrophy was detected in some neurons of the substantia nigra (**Insert 30**). In ALAD + COM group, the cerebral cortex and hippocampus (subiculum, fascia dentate and hilus) showed normal histological structure (**Inserts 31–33**). Focal fine plagues were detected in striatum (**Insert 33**). There was atrophy in some neuronal cells in the substantia nigra (**Insert 35**). In ALAD + COM + PhM group, there was no histopathological alteration in the cerebral cortex, hippocampus (subiculum, fascia dentate and hilus), striatum and substantia nigra (**Insert 36–40**). [The data of the effect of MOR, TML and TMQ on the control is not shown as it is not significant].

Brain tissues of the control group showed normal architecture without abnormal histological changes. However, in the ALAD group, the neuronal cells of the cerebral cortex, as well as the subiculum and fascia dentate of the hippocampus, showed nuclear pyknosis and degeneration. Additionally, in the striatum, eosinophilic plagues were found in isolated foci, and the neuronal cells of the substantia nigra revealed atrophy. All the above histological changes were greatly improved by all the preventive means, whether PhM, MOR, TML, or TMQ. The group treated with phytochemical combination together with PhM, on the other hand, achieved astonishing results of negligible histoarchitecture modifications in brain tissues, particularly the cortex and hippocampus (Figure 2 and Table 1). The data for the effect of MOR, TML and TMQ on the control are not shown as they were not significant. 

### 2.3. Treatment of Rats with MOR, TML, and TMQ Restores Antioxidant Activities and Neurotransmitters Levels in ALAD 

In the ALAD group, AlCl_3_ administration significantly induced oxidative stress by depleting endogenous antioxidants and SOD levels by 78% and 86%, respectively, and enhancing MDA levels by 19-folds in brain tissues. Furthermore, brain neurotransmitters such as DA (19%), 5-HT (36%), and NE (24%), as well as ACHE (562%), were suppressed (*p* < 0.05) in the ALAD group when compared to the control group. In contrast, treatment with a combination of MOR, TML, and TMQ with PhM restored the TAC and SOD levels and produced a significant decrease (*p* < 0.05) in MDA level. Moreover, COM therapy of the phytochemicals with PhM improved (*p* < 0.05) the levels of brain neurotransmitters significantly and averted the elevated levels of ACHE induced by AlCl_3_ administration as compared to the ALAD group (Table 2).

### 2.4. Treatment of Rats with MOR, TML, and TMQ Stimulates the Gene and Protein Expressions of Oxidative Stress Markers: Nrf2 and HO-1 in the Brain Tissues of ALAD 

**Figure 3:**
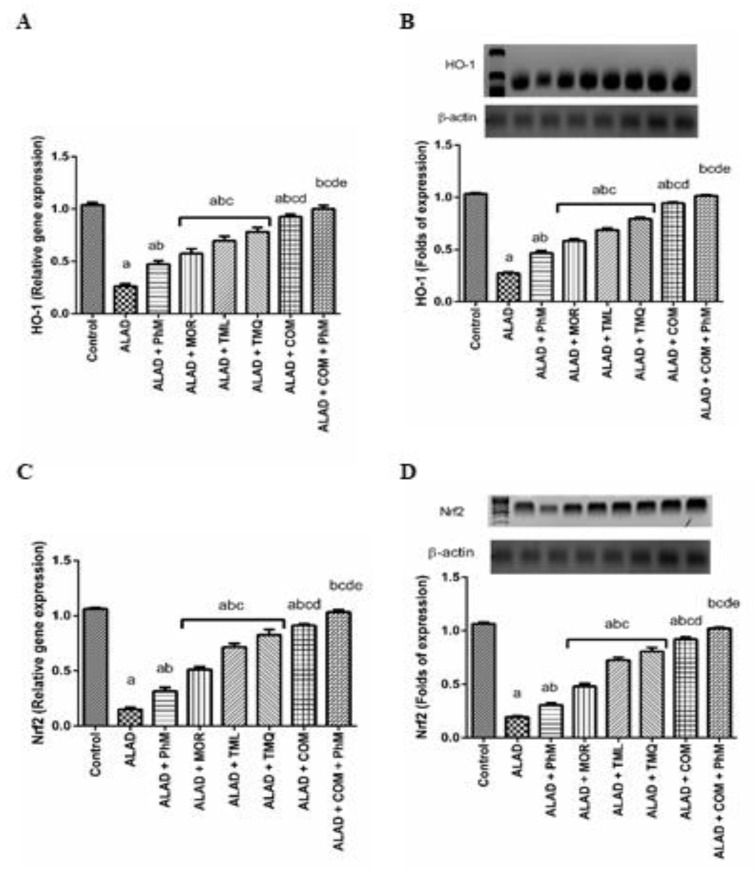
The effect of phytochemicals combination, MOR, TML, and TMQ, with PhM on the gene expression of *HO-1* and *Nrf2* and their protein levels in AD. (**A**) Relative gene expression of *HO-1*, (**B**) Protein expression of HO-1, (**C**) Relative gene expression of *Nrf2*, (**D**) Protein expression of Nrf2. The data are presented as means ± SD (*n* = 7). Significance (a): relative to the control group. Significance (b): relative to the ALAD group. Significance (c): relative to ALAD + PhM group. Significance (d): relative to either ALAD + MOR, ALAD + TML, or ALAD + TMQ group. Significance (e): relative to ALAD + COM group Significance: *p* < 0.05. The data of the effect of MOR, TML and TMQ on the control are not shown as they were not significant.

Alterations in *Nrf2* and *HO-1* levels were observed in the ALAD group since their mRNA and protein expressions were reduced significantly by more than 80% and 70%, respectively, in respect to the control group (Figure 3A–D). However, treatment of the ALAD group with MOR, TML, and TMQ with PhM activated Nrf2/HO-1 signaling (*p* < 0.05) as corroborated by restoring, in part, gene and protein expressions of *Nrf2* and *HO-1* compared to the ALAD group (*p* < 0.05), thus exerting neuroprotective effects. TMQ also has the maximum ameliorating activity for the restoration of the antioxidant biomarkers compared to the effects of MOR and TML. The data of the effect of MOR, TML and TMQ on the control are not shown as they were not significant. 

### 2.5. Phytochemicals with PhM Reduce Neuroinflammatory Biomarkers: TLR4, NF-kB, IL-1β and TNF-α in the Brain Tissues of ALAD 

**Figure 4:**
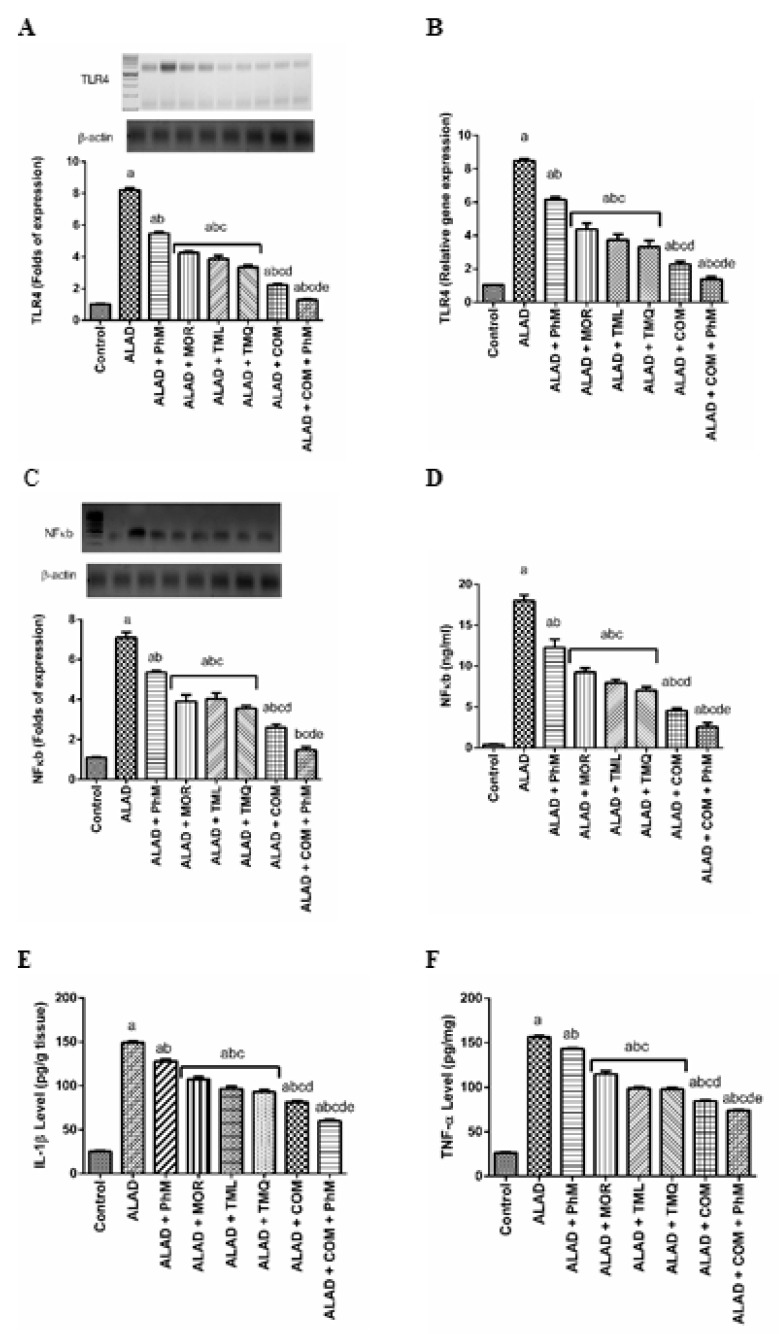
The effect of phytochemicals combination, MOR, TML, and TMQ, with PhM on TLR4 signaling and inflammatory cascade in AD. (**A**) Protein expression of TLR4, (**B**) Relative gene expression of TLR4, (**C**) Protein expression of NF-κb, (**D**) Relative gene expression of NF-κb, (**E**) Protein levels of IL-1β, (**F**) Protein levels of TNF-α. The data are presented as means ± SD (*n* = 7). Significance (a): relative to the control group. Significance (b): relative to the ALAD group. Significance (c): relative to ALAD + PhM group. Significance (d): relative to either ALAD + MOR, ALAD + TML, or ALAD + TMQ group. Significance (e): relative to ALAD + COM group Significance: *p* < 0.05. The data of the effect of MOR, TML and TMQ on the control are not shown as they were not significant.

In the ALAD group, the inflammatory markers; TLR4 and NF-kB, were 8 and 7-times upregulated on the mRNA, as well as on protein levels by 8, 7.4 times, respectively (Figure 4A, C). Moreover, their protein expression levels were also increased. In parallel, TLR4, NF-kB, IL-1β and TNF-α levels were upregulated on the protein level by 9, 18, 8 and 8.3 times, respectively (Figure 4B, D–F). A combination of MOR, TML, and TMQ, significantly repressed the neuroinflammatory cascade induced by AlCl_3_ in ALAD rats. The gene and protein expression of TLR4 was reduced by 84% (*p* < 0.05) after combination therapy along with PhM (Figure 4A, B). Moreover, this combination paved the way for the inflammatory cytokines to be significantly reduced, since IL-1β and TNF-α levels declined by 60% and 52.6% (*p* < 0.05), respectively, with concurrent declines in mRNA level and NF-κB protein expression, when compared with ALAD group (*p* < 0.05) as presented in (Figure 4A–F). TMQ also had a maximum ameliorating activity for the restoration of the neuroinflammatory markers compared to the effect of individual MOR and TML treatments. The data of the effect of MOR, TML and TMQ on the control are not shown as they were not significant. 

### 2.6. A Combination of MOR, TML, and TMQ with PhM Reduces Tissue Injury Biomarker; CHI3L1, and Apoptosis Biomarker; Bax/Bcl-2, and Enhances the Cognitive Biomarker; BDNF in Rat Brain Tissues of ALAD

**Figure 5:**
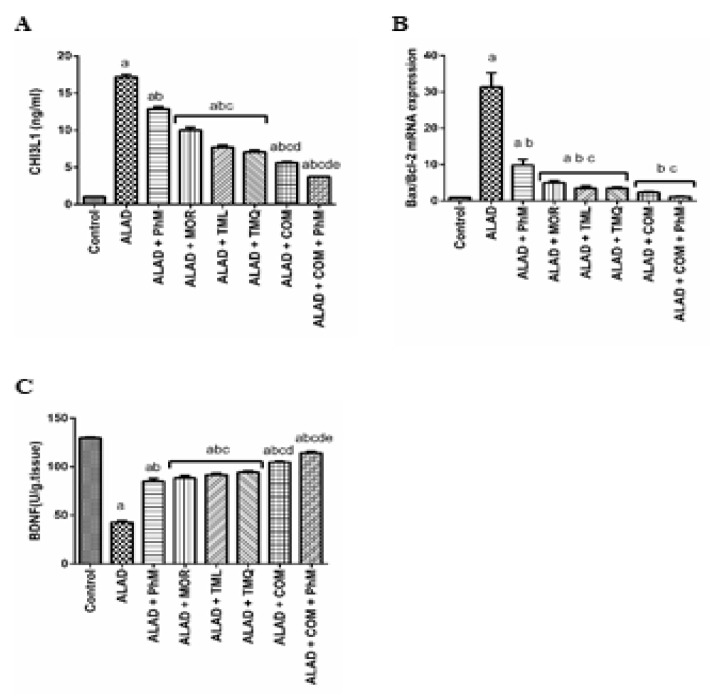
The effect of phytochemicals combination, MOR, TML, and TMQ, with PhM on CHI3L1, BDNF, and apoptosis in AD. (**A**) CHI3L1 levels, (**B**) *Bax/Bcl-2* ratio, and (**C**) BDNF levels. The data are presented as means ± SD (*n* = 7). Significance (a): relative to the control group. Significance (b): relative to the ALAD group. Significance (c): relative to ALAD + PhM group. Significance (d): relative to either ALAD + MOR, ALAD + TML, or ALAD + TMQ group. Significance (e): relative to ALAD + COM group Significance: *p* < 0.05. The data of the effect of MOR, TML and TMQ on the control are not shown as they were not significant.

The inflammatory biomarker of AD, CHI3L1, was evaluated by the ELISA technique. The ALAD group showed a 17-fold elevation in CHI3L1 protein expression (Figure 5A). PhM reduced the elevated level by 23.52%. However, MOR, TML and TMQ significantly reduced its elevated level by 41.18, 52.94 and 56.32%, respectively. Notably, the maximum reduction was obtained by the TMQ treatment. However, a combination MOR, TML, and TMQ, along with PhM had an ameliorating effect for AlCl_3_-induced elevated levels through significant decrease in CHI3L1 protein expression (*p* < 0.05) when compared to the ALAD group. Apoptosis was induced in the ALAD group (elevated *Bax/Bacl-2* mRNA level by about 30-fold) and this effect was halted by a combination therapy with PhM, which caused a reduction in Bax/Bcl-2 mRNA level by 90.63% (Figure 5B). Moreover, the combined phytochemicals significantly upregulated the low level of the cognitive biomarker; BDNF, in the brain tissues (Figure 5C). The data of the effect of MOR, TML and TMQ on the control are not shown as they were not significant.

### 2.7. Treatment of ALAD with Phytochemicals Lowers Potential AD Biomarkers (Aβ Generation, BACE1, APP and p-Tau Levels) in Rat Brain Tissues of ALAD

**Figure 6:**
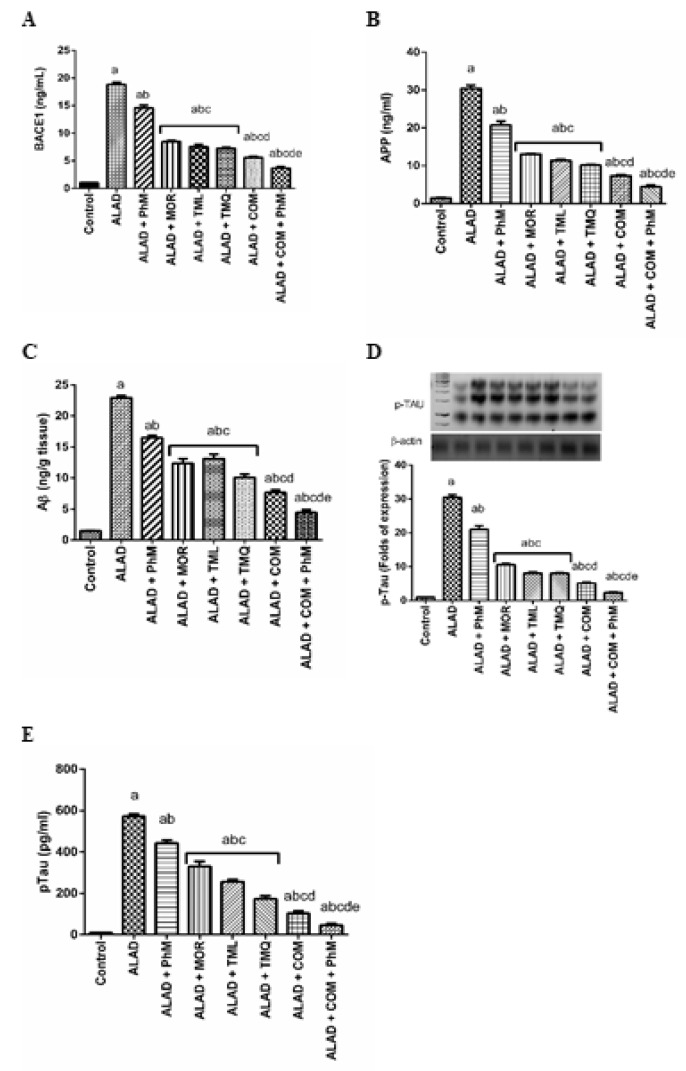
The effect of phytochemicals combination, MOR, TML, and TMQ, with PhM on Aβ aggregation and Tau hyperphosphorylation in AD. (**A**) BACE1 levels, (**B**) APP levels, (**C**) Aβ levels, (**D**) Folds of p-Tau protein expression, (**E**) p-Tau levels. The data are presented as means ± SD (*n* = 7). Significance (a): relative to the control group. Significance (b): relative to the ALAD group. Significance (c): relative to ALAD + PhM group. Significance (d): relative to either ALAD + MOR, ALAD + TML, or ALAD + TMQ group. Significance (e): relative to ALAD + COM group Significance: *p* < 0.05. The data of the effect of MOR, TML and TMQ on the control are not shown as they were not significant.

As illustrated in Figure 6A–E, AlCl_3_ treatment significantly increased the activity of BACE1 and APP levels in brain tissues (20 and 30-fold changes, respectively), which in turn enhanced the level of Aβ in the ALAD group (23.4 times) compared with control groups (*p* < 0.05). Moreover, ELISA and Western blot analyses showed elevated levels of p-Tau, indicating Tau hyperphosphorylation (30.3-fold). However, the combination therapy of MOR, TML, and TMQ in addition to PhM significantly ameliorated Aβ generation and Tau hyperphosphorylation. The levels of BACE1, APP, and Aβ were reduced by 80.3, 85.5, and 80.7%, respectively, and p-Tau was reduced by 92% compared with the ALAD group (*p* < 0.05). TMQ also had the maximum ameliorating activity for the restoration of the AD biomarkers compared to the effects of MOR and TML. The data of the effect of MOR, TML and TMQ on the control are not shown as they were not significant. 

### 2.8. A Combination of MOR, TML, and TMQ with PhM Modulates AD Pathophysiology Biomarker; ApoE4 and its Neuronal Receptor LRP1 in Brain Tissues of ALAD 

**Figure 7:**
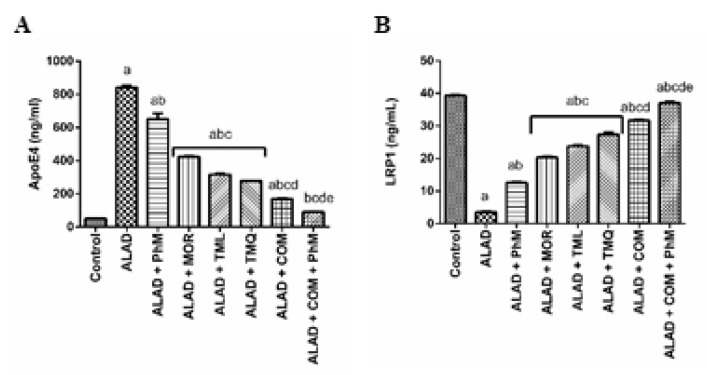
The effect of phytochemicals combination, MOR, TML, and TMQ, with PhM on ApoE4 and LRP1 levels in AD. (**A**) ApoE4 levels and (**B**) LRP1 levels. The data are presented as means ± SD (*n* = 7). Significance (a): relative to the control group. Significance (b): relative to the ALAD group. Significance (c): relative to ALAD + PhM group. Significance (d): relative to either ALAD + MOR, ALAD + TML, or ALAD + TMQ group. Significance (e): relative to ALAD + COM group Significance: *p* < 0.05. The data of the effect of MOR, TML and TMQ on the control are not shown as they were not significant.

It is obvious that AlCl_3_ treatment exacerbated a significant change in the levels of ApoE4 and LRP1 in the ALAD group, as shown in Figure 7A,B. ApoE4 was increased by 16.6-fold, while LRP1, which can endocytose different ligands including ApoE4, Aβ and Tau, was reduced by 91% when compared with the control group. Combination therapy using MOR, TML, and TMQ along with PhM, paved the way for regulating their levels, since this combination with PhM reduced ApoE4 level by 89% compared with the ALAD group and normalized LRP1 level (*p* < 0.05). TMQ also had the maximum ameliorating activity for the restoration of the AD pathophysiology biomarker compared to the effects of MOR and TML. The data of the effect for MOR, TML and TMQ on the control are not shown as they were not significant. 

### 2.9. A Combination of MOR, TML, and TMQ with PhM Regulates Wnt3/β-Catenin/GSK3β Signaling Pathway in Brain Tissues of ALAD 

**Figure 8:**
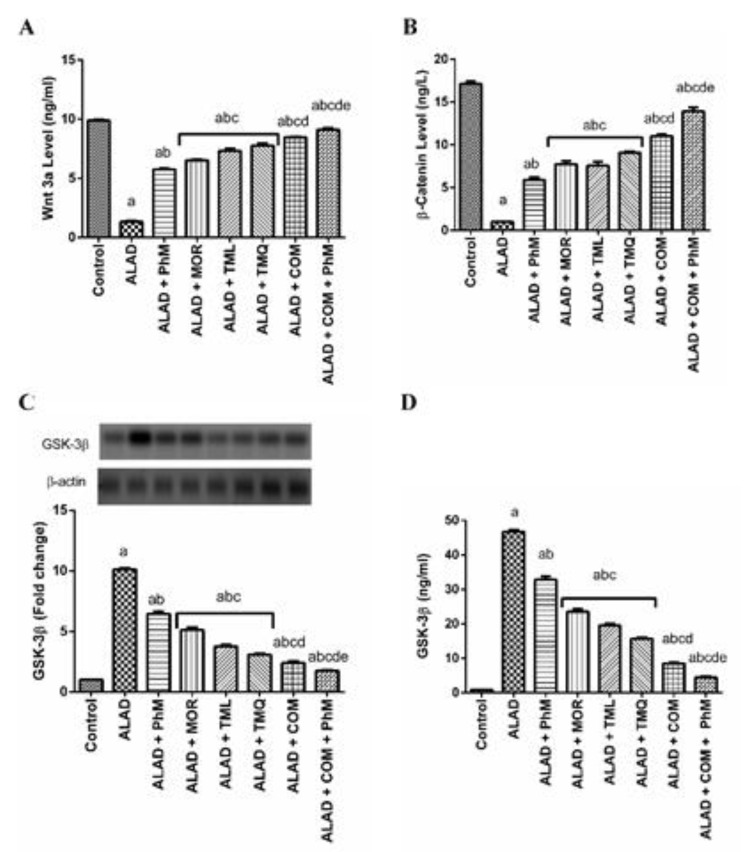
The effect of phytochemicals combination, MOR, TML, and TMQ, with PhM on Wnt3/β-catenin/GSK-3β signaling in AD. (**A**) Wnt3a levels, (**B**) β-catenin level, (**C**) Folds of GSK-3β protein expression, and (**D**) GSK-3β levels. The data are presented as means ± SD (*n* = 7). Significance (a): relative to the control group. Significance (b): relative to the ALAD group. Significance (c): relative to ALAD + PhM group. Significance (d): relative to either ALAD + MOR, ALAD + TML, or ALAD + TMQ group. Significance (e): relative to ALAD + COM group Significance: *p* < 0.05. The data of the effect of MOR, TML and TMQ on the control are not shown as they were not significant.

In the present study, the combination therapy of the phytochemicals, MOR, TML, and TMQ, with PhM markedly suppressed the decrease of both wnt3a and β-catenin that were down-regulated as a result of AlCl_3_ treatment. Meanwhile, GSK3β activity was significantly inhibited by combination therapy. Figure 8A–C shows a significant decrease in Wnt3a and β-catenin levels by 90% and 94%, respectively, with concurrent activation of GSK3β activity by 10-folds as determined by immunoblotting analysis, and by 48-times by ELISA, in comparison with the control group (*p* < 0.05). However, these changes were reversed remarkably with a combination of phytochemicals with PhM compared with the ALAD group (*p* < 0.05). TMQ also had the maximum ameliorating activity for the restoration of the Wnt3/β-catenin/GSK3β signaling pathway compared to the effects of MOR and TML. The data of the effect of MOR, TML and TMQ on the control are not shown as they were not significant.

### 2.10. A Combination of Phytochemicals with PhM Suppreses Inflammasome Activation Biomarkers; NLRP3 and Caspase-1 in Brain Tissues of ALAD

**Figure 9:**
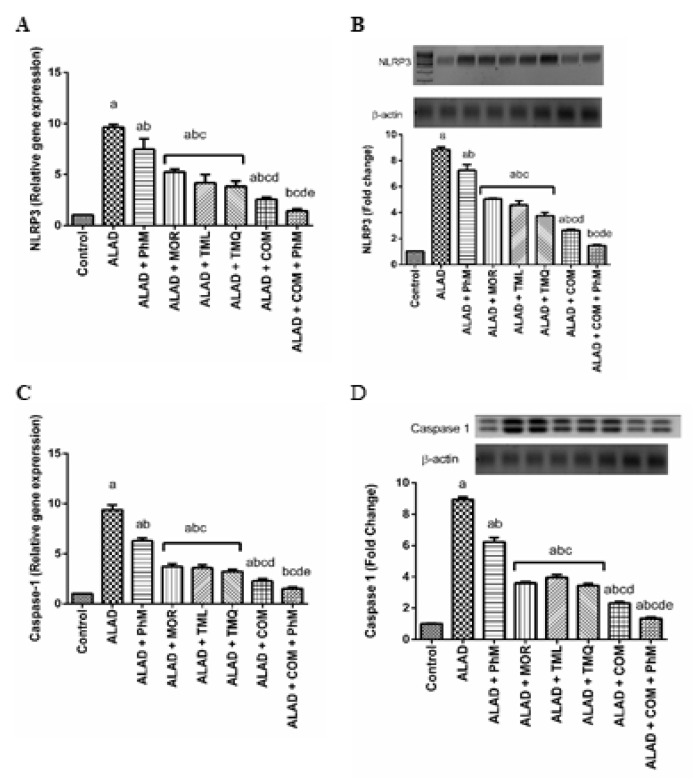
The effect of phytochemicals combination, MOR, TML, and TMQ, with PhM on inflammasome signaling in AD. (**A**) Relative gene expression of NLRP3, (**B**) Folds of NLRP3 protein expression, (**C**) Relative gene expression of caspase-1, and (**D**) Folds of caspase-1 protein expression. The data are presented as means ± SD (*n* = 7). Significance (a): relative to the control group. Significance (b): relative to the ALAD group. Significance (c): relative to ALAD + PhM group. Significance (d): relative to either ALAD + MOR, ALAD + TML, or ALAD + TMQ group. Significance (e): relative to ALAD + COM group Significance: *p* < 0.05. The data of the effect of MOR, TML and TMQ on the control are not shown as they were not significant.

A combination of MOR, TML, and TMQ, along with PhM alleviated the activation of the NLRP3 inflammasome that was up-regulated due to AlCl_3_ treatment, resulting in neuroinflammation as shown in the ALAD group. The mRNA of inflammasome proteins, such as NLRP3 and caspase-1, were estimated by RT-PCR, and their protein levels were determined by Western blotting. As presented in Figure 9A–D, we observed a significant increase (*p* < 0.05) in the mRNA and protein expression of NLRP3 and caspase-1 in the ALAD group on mRNA and proteins levels (9.8 and 9.7-fold respectively) in comparison with the control group (*p* < 0.05). Nevertheless, phytochemicals treatment with PhM exhibited a significant down-regulation (*p* < 0.05) in mRNA and protein expression of NLRP3 and caspase-1 compared with the ALAD group (*p* < 0.05). TMQ also had the maximum ameliorating activity for the restoration of the inflammasome activation biomarkers compared to the effect of MOR and TML. The data of the effect of MOR, TML and TMQ on the control are not shown as they were not significant. 

### 2.11. Molecular Docking of Phytochemicals Used for Treatment of ALAD

The docking study showed that the identified compounds can interact with the active sites of the co-crystallized ligand binding sites in cholinesterase, Cdk5, BACE, glycogen synthase kinase 3 and Keap-1 (Kelch-like ECH-associated protein 1), which control Nrf2 activation. MOR, TMQ, and TML showed differential binding affinities, expressed as docking S-scores (Supplementary Tables S1–S4). MOR showed higher binding scores than TMQ and TML, in that order. The prospective binding interactivity between the protein domains of the co-crystallized ligands is shown in Supplementary Figures S1–S10. In cholinesterase, TML and TMQ showed H-pi and pi-pi interactions with Trp 84 like galantamine, while MOR showed H-pi interaction with Asp 72 like galantamine. MOR and TMQ showed hydrogen bonding with Ser 200. For CDK5, all compounds showed binding with Asn 144 and Val 18 similar to that of the co-crystalized ligand 3O0. In BACE, MOR showed 4 H-bonding interactions with Ser 229 like that of the ligand 66F while TML and TMQ showed pi-H interactions with Ser 10. In GSK, MOR showed H-pi interactions with Tyr 134 and Leu 188 and a strong ionic bond with Lys 85. Keap-1 contains a subset of highly reactive cysteine (Cys) residues. Cys 151 represents one of the major sensors in KEAP1. All compounds showed binding with Cys 151 like that of the co-crystalized ligand D8N. While MOR showed additional H- bond with Ser 103, arene-H bond with Ala 143 and arene-arene bond with Phe 64.

## 3. Discussion

The current study investigated the protective influence of phytochemicals supported by PhM against AD progression induced by AlCl_3_ via exploring their impact on four interconnected axes, namely, Nrf2/HO-1, TLR4/NLRP3, APOE4/LRP1, and Wnt3/β-catenin/GSK-3β pathways that associated with the pathophysiology of AD. Referring to the Nrf2/HO-1 signaling pathway, in most of diseases associated with degenerative brain tissue, including AD, Nrf2 activity was decreased [30]. Such reduced activity leads to mitigation of the oxidative stress damage induction in the brain, which is the pivotal pathological change during the early stage of AD [38,39]. Our results showed the induction of AD by AlCl_3_ administration was associated with a significant induction in oxidative stress due to hampering Nrf2/Ho-1 signaling. This depleted the endogenous antioxidants and SOD levels and enhanced MDA levels in brain tissues. There is a strong correlation between Al and the development of AD. As revealed by our results, Al can cross the blood-brain barrier and deposit in various regions of the brain tissues, which encourages the deposition of Aβ, aggregation of hyperphosphorylated tau, lipid peroxidation, and impairment in learning and memory [26,40]. Increasing the intrinsic antioxidant defense is one of the successful strategies that can prevent oxidative stress-related neuronal pathologies, such as AD [14]. Phytochemicals are promising therapeutics for the treatment of AD due to their functions in inhibiting oxidative stress, neuroinflammation, and mitochondrial dysfunction [41]. The combination of MOR, THL, and TMQ alongside PhM dampened AlCl_3_-induced oxidative stress by raising Nrf2 and HO-1 levels, thus exerting a neuroprotective effect supported by increased catecholamine levels, reduced AChE, and improved behavioral and cognitive functions. The latter was evidenced by improved avoidance response by decreasing the number of the rats’ trials to evade the electric shock on both the first and second days of the CAR test, reduced SAP%, as indicated by the Y-Maze test, and improved learning and spatial memory, as indicated by the results of the MWM test. Different studies have reported that THL, TMQ, and MOR demonstrated an inhibitory effect on AChE activity [36,42,43] which is supported by our docking and experimental studies. TMQ has a significant neuroprotective effect on hippocampal cells of rats, which is mainly correlated with the inhibition of lipid peroxidation after cerebral ischemia [44,45]. It also reduces inflammation caused by oxidative stress [46,47]. Compared to the ALAD group, MOR showed an anti-lipid peroxidation activity [35], reduced the amount of nitrite in the body, and raised the levels of GSH [48,49].

Neuroinflammation is a serious feature of AD pathology [50] that has been attributed to microglial activation accompanied by the release of copious amounts of proinflammatory cytokines, which contributes to neuronal death and degeneration [38]. Because of the ALAD group’s disrupted Nrf2/HO-1 axes and antioxidant status, the inflammatory cascade was triggered by increasing NF-κb expression, thereby increasing the levels of proinflammatory cytokines such as TNF-α and IL-1β, and causing neuroinflammation in AD [38,51,52]. Inflammatory cytokines such as IL-1β are released by microglia and contribute to tau hyperphosphorylation [53]. Moreover, the TLR4/NLRP3 inflammasome is activated by chronic inflammation, leading to neuroinflammation, Aβ accumulation, synapse loss, and neurodegeneration [54]. This is in accordance with our results since chronic administration of AlCl_3_ activates the TLR4/NLRP3 pathway. The activation of the NLRP3 inflammasome contributes to the activation of the inflammatory caspase-1, resulting in the production of proinflammatory cytokines such as IL-1β, which may mediate a variety of local and systemic immune responses [55,56]. Several studies have shown that the NLRP3 inflammasome is closely related to tau pathology [57,58]. Given this, a significant increase in the level of NF-κB was observed in the hippocampus of AD compared to normal control rats, followed by AD-induced neuroinflammation as evidenced by increases in hippocampal TNF-α and IL-1β levels [38]. Additionally, exogenous and endogenous ligands are recognized by the most studied member of the TLR family, TLR4, which responds to inflammation and promotes inflammatory signal transduction via myeloid differentiation factor88 (MyD88) and NF-κB [59]. TLR4 is involved in the regulation of cellular senescence and its expression increases with chronic exposure to proinflammatory cytokines during aging, which is known as inflammaging [60,61]. Therefore, the TLR4/NLRP3 pathway has a significant role in the progression of AD. On the contrary, inhibition of TLR4 can ameliorate learning and memory impairment, diminish Aβ deposition, and suppress neuronal apoptosis, thus providing neuroprotection in an AD mouse model [62]. Recent research has shown that phytochemicals in food can protect neurons from neurological diseases caused by the NLRP3 inflammasome by blocking the TLR4 axis pathway signaling pathway [63,64,65]. These data support our results, which showed a combination of MOR, TML, and TMQ was significantly repressed the neuroinflammatory and pro-apoptotic cascades that were induced by AlCl_3_ treatment. The expression of TLR4 and NLRP3 was reduced after phytochemical combinations in addition to PhM when compared with the ALAD group. Furthermore, this combination therapy paved the way for inflammatory cytokines to be significantly reduced, as indicated by reduction in IL-1β and TNF-α levels and NF-κB protein expression.

Viewing the ApoE4/LRP1 axis, which has been connected to the progression of AD, was next on our list. Reduced LRP1 levels have been reported during aging and AD development [66]. LRP1 regulates many Aβ-degrading enzymes and cellular degradation pathways in astrocytes, which are required for clearing brain Aβ [67,68]. Additionally, LRP1 mediates tau internalization and degradation due to the binding affinity of tau and the tau microtubule-binding domain to LRP1 [69]. These receptors are known to bind to other diversified extracellular ligands, including ApoE, the primary component of lipoproteins in the human brain, and it is a genetic risk factor for AD. ApoE4 has been implicated in numerous processes, including crosstalk with Aβ, and has been shown to influence lipid metabolism and inflammation [70]. Unfortunately, because ApoE4 has a higher binding affinity for LRP1, LRP1-mediated tau uptake and Aβ clearance are inhibited [68]. This confirms that the Aβ deposition in AD patients is more abundant in ApoE4 carriers in comparison with non-carriers [71,72]. In an in vitro model using PC12 cells transfected with the human ApoE4 gene, Al decreased LRP1 proteins and cell survival with coinciding elevation of Aβ content in PC12 cells in a dose-dependent manner [73]. In the current study, it was obvious that AlCl3 treatment exacerbated a significant change in the levels of ApoE4 and LRP1 in the ALAD group. ApoE4 was increased while LRP1 levels were decreased when compared to the control group. This supports the idea that chronic exposure to Al intoxication can provoke AD progression through engagement of theApoE4/LRP1 axis. However, the combination therapy along with PhM interferes with the effect of AlCl_3_ in altering the expression of ApoE4 and LRP1. A similar finding was reported by Ismail et al., where TMQ treatment decreased Aβ levels by modulating APP processing, upregulating LRP1, and downregulating BACE1 [74]. Phytochemicals can modify the structure of ApoE4 and amend the pathogenic effects of ApoE4-mediated oxidative stress and AD progression [75].

Finally, dysregulation of Wnt3/β-catenin/GSK-3β axis is linked to a wide range of illnesses, including cancer, kidney disease, bone problems, and neurodegenerative diseases [76]. GSK-3β dysregulation is associated with the etiology of AD and Aβ-induced neurotoxicity. Thus, inhibiting GSK-3β has benefits against neurodegeneration and AD [77]. Intriguingly, combining the MOR, TML, and TMQ together with PhM compensated the downregulation of Wnt3a and β-catenin caused by AlCl_3_ treatment. Meanwhile, GSK-3β activity was significantly inhibited due to combination therapy with PhM. This confirms our molecular docking results that indicate the high binding affinity of MOR, TML, and TMQ to GSK-3β. Therefore, the activation of Wnt3/β-catenin signaling inhibits Aβ production and tau protein hyperphosphorylation [78] and upregulates BDNF, which is necessary for synaptic plasticity and restoring learning and memory impairments [38,61]. Our findings show that phytochemicals such as MOR, TML, and TMQ supported by PhM, have a potential role in precluding the neurodegenerative effects and AD progression. This occurs by modulating different axes that are involved in AD, in particular the Nrf2/HO-1, TLR4/NLRP3, APOE4/LRP1, and Wnt3/β-catenin/GSK-3β signaling pathways.

## 4. Materials and Methods

### 4.1. Drugs and Chemicals

Aluminum chloride (AlCl_3_) (CAS Number 7446-70-0, Morin (MOR) (CAS Number 480-16-0), Thymoquinone (TMQ) (CAS Number 490-91-5), and Thymol (THL) (CAS Number 89-83-8) were purchased from Sigma-Aldrich Co. Inc. (St. Louis, MO, USA). They were dissolved in distilled water.

### 4.2. Animals and Induction of AD-Like Rat Model

All animal treatment methods and procedures were approved by the Ethical Research Project Committee in the Faculty of Pharmacy, Al-Azhar University, Cairo, Egypt (Protocol approval number 320/2022). All procedures and experiments were performed in accordance with the relevant guidelines and regulations for the Care and Use of Laboratory Animals published by the National Institutes of Health. Adult healthy male Dawley rats from 8–10 weeks-old and weighing 300–320 g were utilized in this experiment. The animals were purchased from the Nile Co. for Pharmaceuticals and Chemical Industries (Cairo, Egypt) and were entrained under controlled-laboratory conditions (temperature 24–26 °C, 12 h light-dark cycles, with free access to water. Rats were kept in polycarbonate cages padded with paper in an individual manner and covered by stainless steel wire (three rats/cage).

### 4.3. Experimental Design

The rats were randomly allocated into eight groups with 10 rats each as follows. Group 1: the rats administered distilled water (i.p., daily for 5 weeks) and considered as a control. Group 2: AlCl_3_-induced Alzheimer’s disease (ALAD) by administering rats with 70 mg/kg AlCl_3_, i.p., daily for 5 weeks) as reported [62]. Group 3: ALAD + PhM. Group 4: ALAD + MOR by administering AlCl_3_ once a week for 5 weeks and 20 mg/kg of MOR every day by mouth [39]. Group 5: ALAD + TML by administering AlCl_3_ in addition to a daily oral administration of TML (30 mg/kg) [63]. Group 6: ALAD + TMQ by given AlCl_3_ along with 10 mg/kg of TMQ every day by mouth [64]. Group 7: ALAD + COM by giving rats AlCl_3_ weekly with a daily administration of a combination therapy of MOR, TML and TMQ. Group 8: animals receiving ALAD + COM + PhM by administering rats AlCl_3_ once a week as well as a daily dose of MOR and TML in addition to PhM. 

At the end of the study, after five weeks, behavioral tests were used to evaluate the extent of spatial recognition and memory impairment.

In this study, an AD-like model was attained by administration of AlCl_3_. It was dissolved in distilled water and administered daily (70 mg/kg, i.p) for five weeks as previously described [65]. The weight of animals was recorded every week and neurodegeneration was assessed by behavioral tests. 

### 4.4. Physical and Mental Activity Study

Physical and mental activities (PhM) were used as a therapeutic tactic to decrease the neurodegeneration induced by AlCl_3_ administration. PhM was achieved twice weekly by subjecting animals to forced swimming test and Y-maze on two different days/week for five weeks [66]. 

### 4.5. Behavioral Tests

#### 4.5.1. The Conditioned Avoidance Response (CAR) Test

The CAR test was used to assess learning ability and memory consolidation in highly stressful conditions [67]. As previously described [68], a special wooden box apparatus with five interconnected chambers using movable glass with four floors powered by a stimulator set to 50 volts and 25 pulses/second was used, while the fifth chamber’s floor was made of glass (safety area). Rats were trained the day before the experiment. The training consisted of 5 s of auditory stimuli (conditioned stimulus) followed by 5 s of foot shock. For two days, the same animals were tested repeatedly. The number of post-treatment trials (on the first and second days) to reach the safety area within 5 s of the conditioned stimulus before starting the electric shock was calculated for each rat.

#### 4.5.2. Y-Maze Spontaneous Alternation (SAP) Test

SAP can reflect spatial working memory, a type of short-term memory [69]. As previously described [70], a black wooden Y-maze with a symmetrical triangular central area with three arms labelled A, B, or C was used. Briefly, rats were placed at the rim of one arm and allowed to roam freely through the maze for 8 min. The entries were counted when the rat’s hind paws were completely inside the arm. SAP was calculated using the following equation based on the number of alternations and total arm entries: SAP (%) = [number of alternations/(total arm entries-2)] × 100.

#### 4.5.3. Morris Water Maze (MWM) Testing

To assess spatial learning and memory, the Morris water maze test was used as reported [71]. Briefly, a circular water tank (60 cm in height and 150 cm in diameter) was filled with tap water to half full. A non-toxic white paint was added to the water to make it opaque. The pool was virtually divided into equal four quadrants (north, south, east, and west). At a fixed location in the center of one of the quadrants, a 10 cm diameter escape platform was hidden 2 cm below the surface of the water. The animals were given 60 s to find the hidden platform and 20 s to rest on it before the next trial began. If they had waited 60 s before finding the platform, they were gently placed on it and allowed to rest for 20 s. The time it took to find the platform (escape latency) was recorded. On the fourth day, the opaque water was replaced with clear water, and a probe test was performed by removing the platform and allowing the rats to swim freely for 60 s. The amount of time spent in the target quadrant was tracked.

### 4.6. Assembling and Preparation of Tissue Samples

At the end of the experiment, and after behavioral assessment, blood samples were assembled from the retro-orbital vein under light anesthesia. Animals were sacrificed under general anesthesia using an overdose of ketamine (100 mg/kg) [72]. Brain tissues were removed immediately after being sacrificed and washed by cold saline. Three samples per each group were used for histopathological examination after being kept in 10% formaldehyde. The rest of the tissues were used for biochemical tests, ELISA, Western blotting, and real-time PCR (RT-PCR) analyses. 

### 4.7. Histopathological Evaluation

As reported [73,74,75,76,77,78], the animals were rinsed with isotonic NaCl solution under deep anesthesia. Afterwards, they were diffused with 1% formaldehyde through the hearth. We then separated their heads, excised their brains, and put them directly on ice. We then sliced their brains both sagittally and coronally. We then fixed all of the slices in 1% formaldehyde for two days. Fixed slices were stained with standard hematoxylin–eosin, air dried and viewed under a light microscope and photomicrographs with a total magnification of 100X and 400X.

### 4.8. Preparation of Tissue

As previously described [79,80,81,82,83,84], the hippocampal cortex tissues were quickly isolated and homogenized in ice-cold Tris-HCl 50 mM (10% *w*/*v*) supplemented with 300 mM sucrose (pH 7.4) and immediately stored at −80 °C until use.

### 4.9. Biochemical Analyses

#### 4.9.1. Colorimetric Analysis

As previously described [85,86,87], the tissue homogenates were tested for oxidative stress biomarkers in the brain tissues. Total antioxidant capacity (TAC), superoxide dismutase (SOD), and malondialdehyde (MDA) were evaluated by commercially available colorimetric assay kits (Biodiagnostic, Cairo, Egypt). Additionally, the AChE content in brain tissue homogenate was determined colorimetrically at wave lengths 660 nm, 540 nm and 532 nm for TAC, SOD and MDA respectively.

#### 4.9.2. Fluorometric Assays

Fluorometric analysis was used to assess the alteration in normal levels of brain monoamine neurotransmitters such as dopamine (DA), norepinephrine (NE), and serotonin (5-HT). The fluorometric analysis was determined using a previously described method [88]. Briefly, monoamines were first oxidized to their “adrenochromes,” and then rearranged to their “adrenolutins,” which were then detected fluorometrically (FLU) with samples at λex/λem 320/385 nm, 385/485 nm, and 360/470 nm for DA, NE, and 5-HT, respectively. The fluorescence of standard solutions was used to determine concentrations as nanograms per gram of fresh tissue.

#### 4.9.3. Enzyme-Linked Immunosorbent Assay (ELISA)

The enzyme-linked immunosorbent analysis (ELISA) technique was employed for estimating inflammatory markers such as GSK3β, IL-1β and TNF-α, using ELISA kits (Ray Biotech, Inc. Cat No: IQR-IL1b and My BioSource, Inc., San Diego, CA, USA Cat No: MBS175904, respectively) in brain tissue homogenate:. Additionally, ELISA kits (MyBioSource, Inc., San Diego, CA, USA) were used to estimate the biomarkers of cognition and the extent of neurodegeneration, including APP, Aβ, BDNF, BRCE1, pTau, NF-κ, Wnt3a, CHI3L1, β-catenin, ApoE4, and LRP1

#### 4.9.4. Western Blotting Assay

Protein expression of Nrf2, HO-1, NF-κB, NLRP3, caspase-1, TLR4, p-tau and p-GSK3β was detected by immunoblotting. Briefly, samples were homogenized in RIPA buffer containing protease inhibitors, then centrifuged. The supernatant was resolved on an SDS-PAGE gel (7%) and proteins were transferred to nitrocellulose membranes. Before probing with the primary antibodies for the targeted proteins, the membranes were blocked by non-fat dried milk in TBST (0.05% Tween-20 Tris-buffered saline) for two hours at room temperature. Then, the membranes were incubated overnight at 4 °C with antibodies for p-tau and p-GSK3 (dilution 1:1000; Thermo Fisher Scientific, Waltham, MA, USA), monoclonal antibodies for Nrf2, HO-1, and TLR4 (1:300, Santa Cruz Biotechnology, Inc, TX, USA), polyclonal antibodies for NF-B and NLRP3 (1:300, My BioSource, Inc., San Diego, CA, USA) and caspase 1 (dilution 1:400; Thermo Fisher Scientific, Waltham, MA, USA), as well as rabbit polyclonal antibodies for β-actin (1:1000). The protein-antibody complex was visualized using a secondary horseradish peroxidase-conjugated anti-rabbit IgG antibody (1:25,000, Bio-Rad, Hercules, CA, USA). A CCD camera-based imager was used to capture the protein signals. Using image analysis software, the ChemiDoc MP imager was used to read the band intensities of the target proteins against the control sample β-actin.

#### 4.9.5. Real-Time Quantitative Polymerase Chain Reaction (RT-qPCR)

Transcripts of *Bcl-2*/*Bax*, *caspase*-1, *NLRP3*, *HO-1*, *Nrf2*, *TLR4*, and the housekeeping gene (β-actin), were assessed in rat brain tissue by RT-qPCR using Applied Biosystems step one plus equipment. Total RNA was isolated using a Qiagen tissue extraction kit (Qiagen, Germantown, MD, USA) according to the manufacturer’s instructions. Reverse transcription of the extracted mRNA was performed by utilizing a sense fast cDNA synthesis kit (Cat No. BIO-65053). Data analyses were performed using an Applied Biosystem with software version 3.1 (StepOne™, Waltham, MA, USA). The sequence of primer sets is shown in Table 3.

### 4.10. Molecular Docking Study

Molecular modeling and visualization processes were performed within choline esterase, Cdk5, BACE and glycogen synthase kinase 3 of the PDB codes: 1DX6 [89], 3O0G [90], 7D2V [91], 5HLP [92] and 6FFM [93]. First, compounds were prepared with the standard protocol designated in Molecular Operating Environment 2019 (MOE 2019.0102, 2020; Chemical Computing Group, Montreal, QC, Canada). The compound structures’ energies were minimized using an MMF94FX Forcefield with a gradient RMSD of 0.0001 kcal/mol. Afterwards, the protein structure was prepared using the MOE preparation protocol. The docking study was validated by redocking the co-crystallized ligands into their binding sites using the same set of parameters as described above. Afterwards, the ligands were docked into the binding site using the alpha triangle matching placement method. Refinement was carried out using forcefield and scored using the affinity dG scoring system. The resulting docking poses were visually inspected, and the pose of the lowest binding free energy value and that of RMSD values less than 2 Å was considered.

### 4.11. Statistical Analysis

Data are expressed as mean ± S.E.M. Multiple comparisons were performed using one-way ANOVA followed by Tukey Kramer as a post hoc test. All statistical analysis and graphing were done performed using GraphPad Prism Software Ver. 5 (ISI^®^, USA). The level of significance was considered at *p* < 0.05.

## 5. Conclusions

Our findings show that TMQ has superior activity for improving learning and memory, and all of the biomarkers such as antioxidant, neuroinflammatory, inflammatory, AD, AD pathophysiology and inflammasone activation biomarkers, compared to MOR and TML. Moreover, our findings show that the administration of a combination of morin, thymol and thymoquinone along with physical and mental activity has an ameliorating effect on the Alzheimer’s disease by inhibiting oxidative stress, inflammatory and apoptosis pathways. These findings offer a novel view for using phytochemicals as neuroprotective agents.

## Figures and Tables

**Table 1:** The histopathological score for the cerebral cortex and hippocampus. The severity of histopathological alterations in rat brain areas is indicated in different experimental groups.

Group		Control	ALAD	ALAD + PhM	ALAD + MOR	ALAD + TML	ALAD + TMQ	ALAD + COM	ALAD + COM + PhM
**Nuclear pyknosis and degeneration**	**Cerebral cortex**	-	+++	-	-	++	+	-	-
	**The subiculum and fascia dentate of the hippocampus**	-	+++	-	++	-	-	-	-
**Focal eosinophilic plagues in the striatum**		-	+++	+++	+	+	-	+	-
**Atrophy in the neuronal cells of the substantia nigra**		-	+++	+++	+	-	+	+	-

Severe: +++; Moderate: ++; Mild: +; Nil: -.

**Table 2:** The effect of phytochemical combinations, MOR, TML, and TMQ, with PhM on oxidative stress, neurotransmitter decline and ACHE elevation induced by AlCl_3_ administration in rats for five weeks (70 mg/kg/day, i.p.).

.	Control	ALAD	ALAD + PhM	ALAD + MOR	ALAD + TML	ALAD + TMQ	ALAD + COM	ALAD + COM + PhM
**TAC** *(mmol/g)*	45.3 ± 1.2	9.7 ± 1.3 ^a^	14.4 ± 1.0 ^ab^	24.3 ± 1.4 ^abc^	25.5 ± 1.7 ^abc^	25.7 ± 1.3 ^abc^	30.7 ± 1.0 ^abcd^	33.7 ± 2.1 ^abcde^
**SOD** (*U/g*)	5.9 ± 0.4	0.8 ± 0.04 ^a^	2.5 ± 0.08 ^ab^	3.1 ± 0.23 ^abc^	3.3 ± 0.3 ^abc^	3.8 ± 0.08 ^abc^	4.4 ± 0.4 ^abcd^	5.8 ± 0.1 ^bcde^
**MDA** (*nmol/g*)	5.5 ± 0.6	104.4 ± 7.5 ^a^	85.4 ± 4.9 ^ab^	72.7 ± 2.3 ^abc^	67.0 ± 3.6 ^abc^	56.7 ± 2.7 ^abc^	41.4 ± 3.4 ^abcd^	25.3 ± 3.7 ^abcde^
**DA** (*ng/g*)	71.2 ± 1.9	13.6 ± 1.9 ^a^	23.7 ± 2.5 ^ab^	40.0 ± 0.2 ^abc^	45.3 ± 0.5 ^abc^	48.8 ± 0.8 ^abc^	53.5 ± 1.0 ^abcd^	58.1 ± 2.4 ^abcde^
**5-HT** (*ng/g*)	11.8 ± 1.0	4.2 ± 0.5 ^a^	6.0 ± 0.4 ^ab^	7.3 ± 0.5 ^abc^	7.9 ± 0.6 ^abc^	8.7 ± 0.4 ^abc^	10.0 ± 0.4 ^abcd^	11.2 ± 0.9 ^bcde^
**NE** (*nmol/g*)	737.2 ± 4.6	176.6 ± 8.2 ^a^	258.0 ± 8.5 ^ab^	390.7 ± 14.8 ^abc^	445.5 ± 7.3 ^abc^	470.1 ± 12.3 ^abc^	533.5 ± 26.6 ^abcd^	594.4 ± 6.4 ^abcde^
**ACHE** (*ng/g*)	11.8 ± 1.1	66.1 ± 1.9 ^a^	49.3 ± 3.0 ^ab^	24.8 ± 0.4 ^abc^	22.4 ± 1.9 ^abc^	22.1 ± 1.8 ^abc^	17.1 ± 1.0 ^abcd^	13.8 ± 0.1 ^bcde^

The effect of phytochemical combinations, MOR, TML, and TMQ, with PhM on oxidative stress, neurotransmitters decline, and ACHE elevation induced by AlCl_3_ administration for five weeks (70 mg/kg/day, i.p.). The data are presented as means ± SD (*n* = 7). Significance (a): relative to the control group. Significance (b): relative to the ALAD group. Significance (c): relative to ALAD + PhM group. Significance (d): relative to either ALAD + MOR, ALAD + TML, or ALAD + TMQ group. Significance (e): relative to ALAD + COM group Significance: *p* < 0.05. The data of the effect of MOR, TML and TMQ on the control are not shown as they were not significant].

**Table 3:** List of primer sequence sets used for RT-qPCR analysis in rat tissues.

Gene	The Primer Pair Sequence	Gen ID	Number of Base Pairs
***Bax***	**F:** 5′-CACGTCTGCGGGGAGTCA-3’ R: 5’-TAGGAAAGGAGGCCATCCCA-3’	**NM_017059**	566 bp
***Bcl-2***	**F:** 5′-CATCTCATGCCAAGGGGGAA-3’ R: 5′-TATCCCACTCGTAGCCCCTC- 3’	**NM_016993**	284 bp
***TLR4***	**F:** 5′-TCAGCTTTGGTCAGTTGGCT-3’ R: 5′-GTCCTTGACCCACTGCAAGA-3’	**NM_019178**	692 bp
***HO-1***	**F:** 5′-CACCAGCCACACAGCACTAC-3′ R: 5′-CACCCACCCCTCAAAAGACA-3′	**NM_012580**	1042 bp
***Nrf2***	**F:** 5′-CTCTCTGGAGACGGCCATGACT-3′ R: 5′-CTGGGCTGGGGACAGTGGTAGT-3′	**NM_031789**	145 bp
***NLRP3***	**F:** 5′-TGCATGCCGTATCTGGTTGT-3′ R: 5′-ACCTCTTGCGAGGGTCTTTG-3′	**NM_001191642**	391 bp
***Caspase-1***	**F:** 5′-GAACAAAGAAGGTGGCGCAT-3′ R: 5′-GAGGTCAACATCAGCTCCGA-3′	**NM_012762**	202 bp
***β-actin***	**F:** 5′-CCGTAAAGACCTCTATGCCA- 3’ R: 5′-AAGAAAGGGTGTAAAACGCA- 3’	**NM_031144**	299 bp

## Data Availability

Data are available on request from corresponding author.

## Supplementary Materials

The following supporting information can be downloaded at: https://www.mdpi.com/article/10.3390/ph15081008/s1, Figures S1–S5: 2D docking poses of compounds; Figures S6–S10: 3D docking of compounds; Table S1. Receptor interaction of compounds A–C; Table S2. Receptor interaction of compounds **1**–**3** and the ligand 3O0; Table S3. Receptor interaction of compounds **1**–**3** and the ligand 66F; Table S4. Receptor interaction of compounds **1**–**3** and the ligand 65A.

## Abbreviations

ALAD	Aluminum chloride-induced Alzheimer’s disease
AD	Alzheimer’s disease
Aβ	Amyloid beta
APP	Amyloid precursor protein
ApoE4	Apolipoprotein E variant 4
BACE1	Beta-site amyloid precursor protein cleaving enzyme 1
BDNF	Brain-derived neurotrophic factor
CAR	Conditioned avoidance response
DAMPs	Damage-associated molecular patterns
*i.p*	Intraperitoneal
Keap-1	Kelch-like ECH-associated protein 1
LRP1	Low-density lipoprotein receptor-related protein-1
MOR	Morin
NFκB	Nuclear factor kapa B
NFTs	Neurofibrillary tangles
MWM	Morris water maze testing
Nrf2/HO-1	Nuclear factor erythroid 2-related factor 2/hemoxygenase-1
PhM	Physical and mental activities
SAP	Spontaneous alternation percentage
THL	Thymol
TMQ	Thymoquinone
TLR-4	Toll-like receptor-4
