# Supplementary material for: Neuroprotective Effects of Phytochemicals against Aluminum Chloride-Induced Alzheimer’s Disease through ApoE4/LRP1, Wnt3/β-Catenin/GSK3β, and TLR4/NLRP3 Pathways with Physical and Mental Activities in a Rat Model
Source: Pharmaceuticals (Basel). 2022 Aug 17;15(8):1008. doi: 10.3390/ph15081008 (PMC9416484; doi:10.3390/ph15081008)
Supplement: Supplementary file 1 [file pharmaceuticals-15-01008-s001.zip › pharmaceuticals-1819839-supplementary.pdf]

Supplementary materials:

1) Figures

Figure S1. 2D docking poses of compounds (A) MOR (B) TML (C) TMQ (D) galantamine in-to the galantamine binding site in choline esterase PDB: 1DX6.

Supplementary data S1

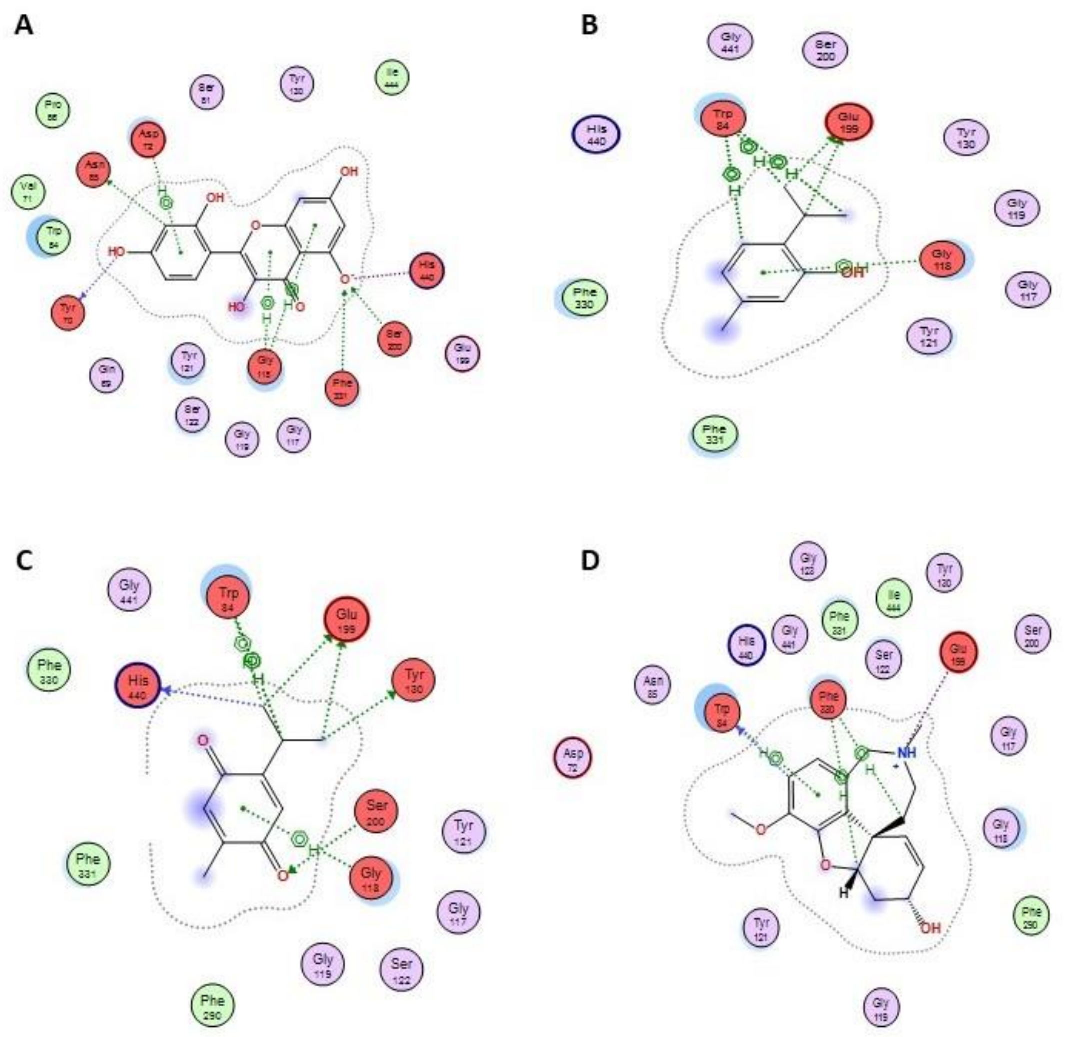

Figure S2. 2D docking poses of compounds (A) MOR (B) TML (C) TMQ (D) the ligand 3O0 into the 3O0 binding site in CDK5 PDB: 3O0G

Supplementary data S2

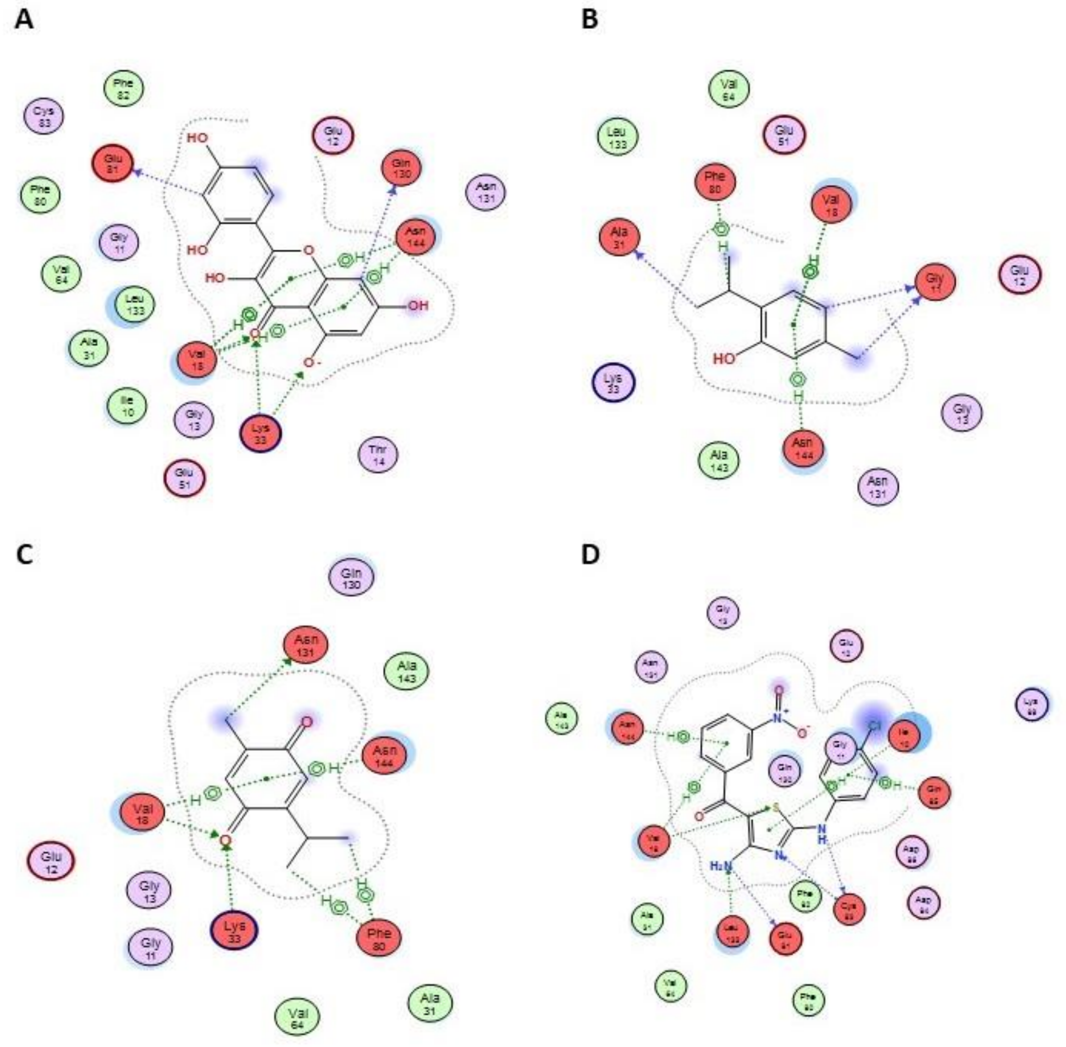

Figure S3. 2D docking poses of compounds (A) MOR (B) TML (C) TMQ (D) ligand 66F into the 66F binding site in BACE 1: 7D2V.

Supplementary data S3

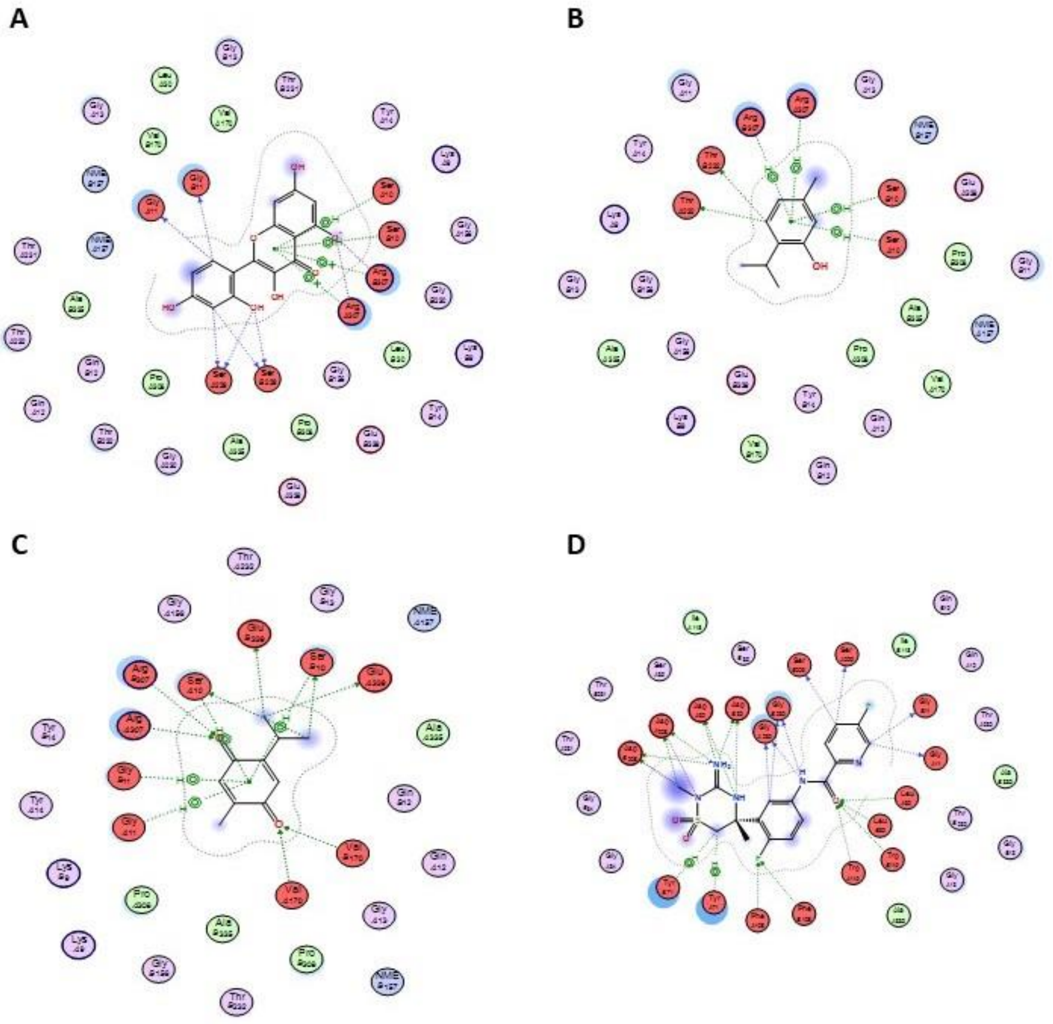

Figure S4. 2D docking of compounds (A) MOR (B) TML (C) TMQ (D) the ligand 65A into the 65A binding site in the glycogen synthase kinase 3 PDB: 5HLP.

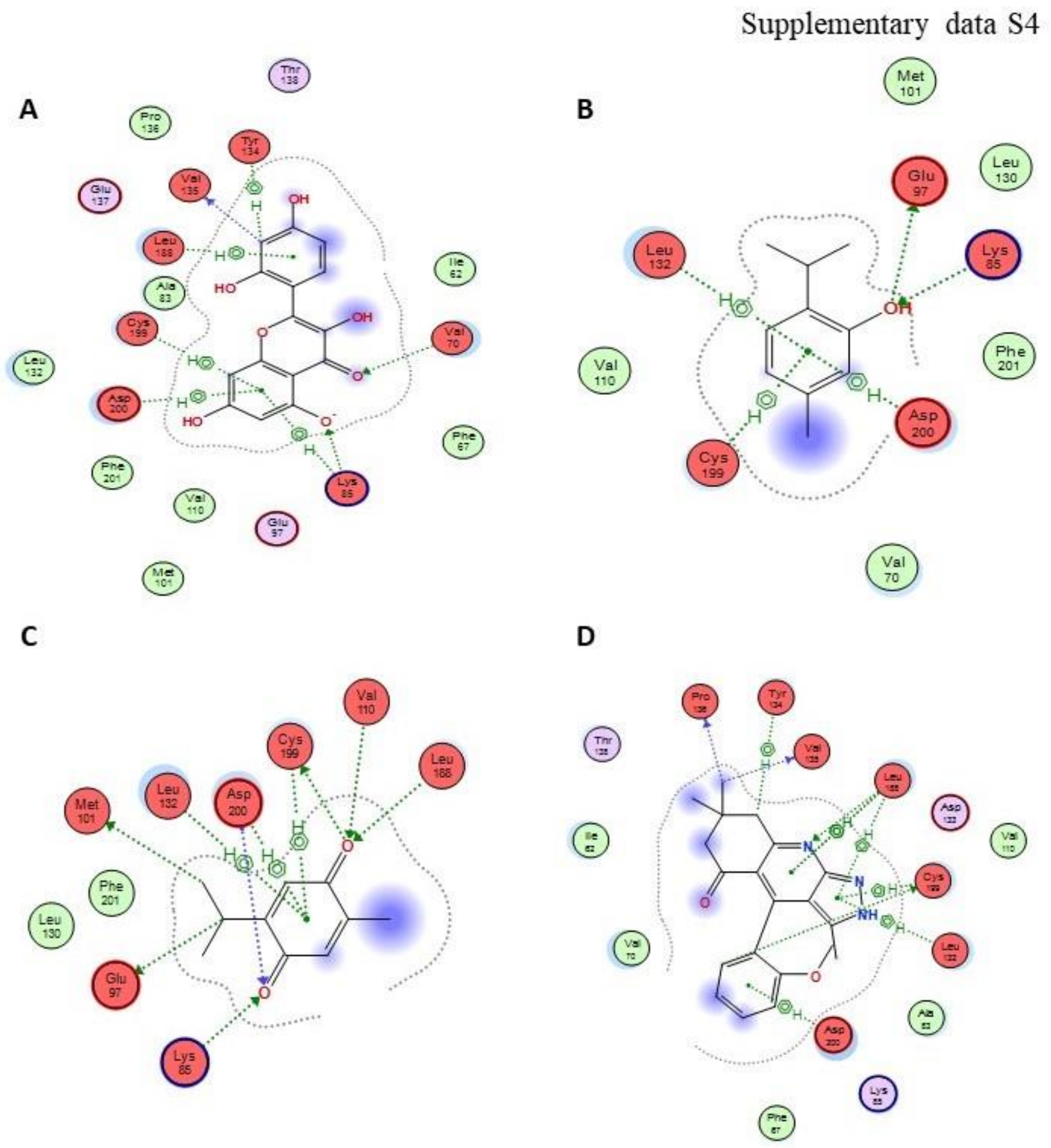

Figure S5. 2D docking of compounds (A) MOR (B) TML (C) TMQ (D) the ligand D8N into the D8N binding site in Keap1/5 complex (PDB ID 6FFM).

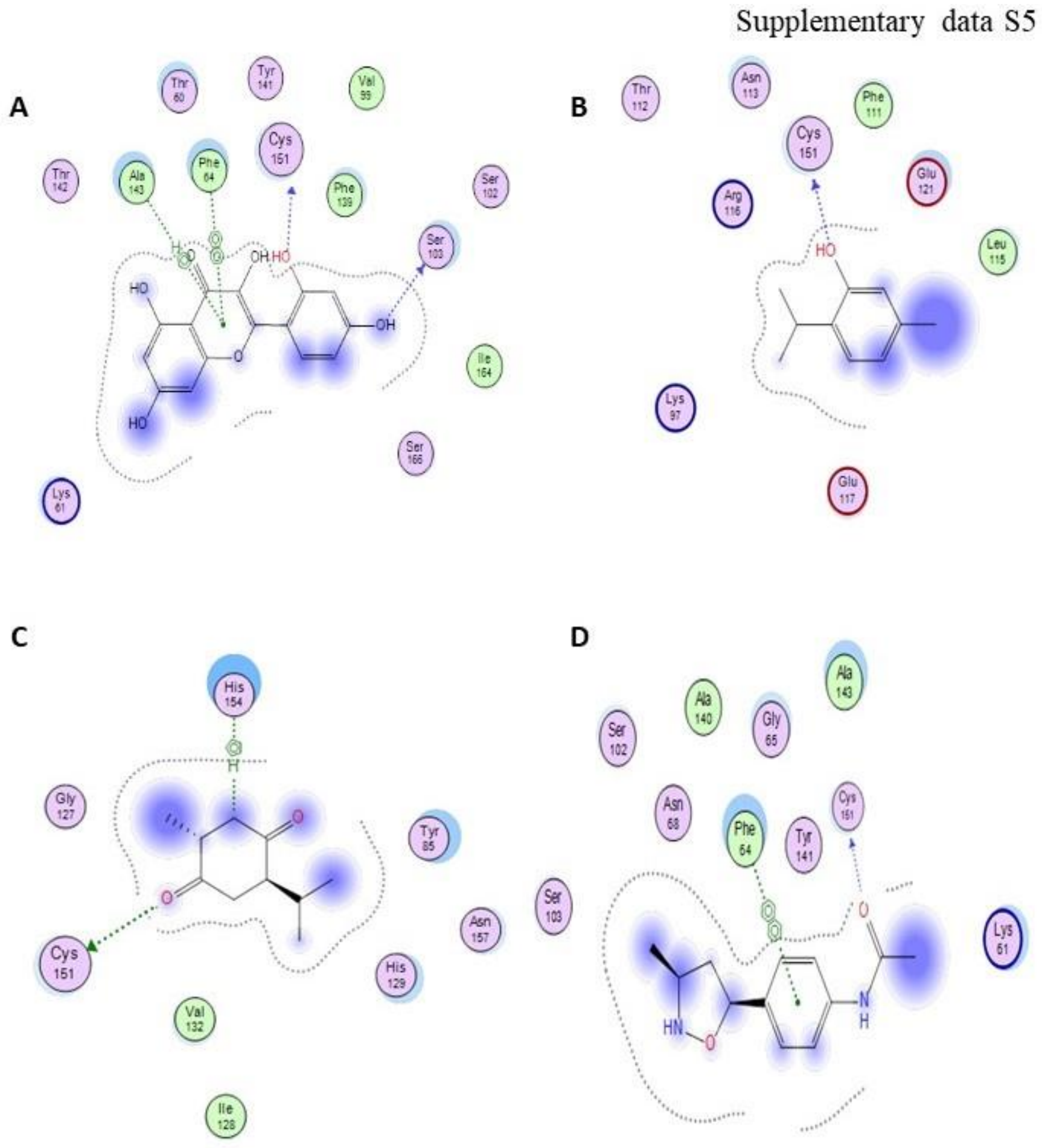

Figure S6. 3D docking poses of compounds (A) MOR (B) TML (C) TMQ (D) galantamine into the galantamine binding site in choline esterase PDB: 1DX6.

Supplementary data S6

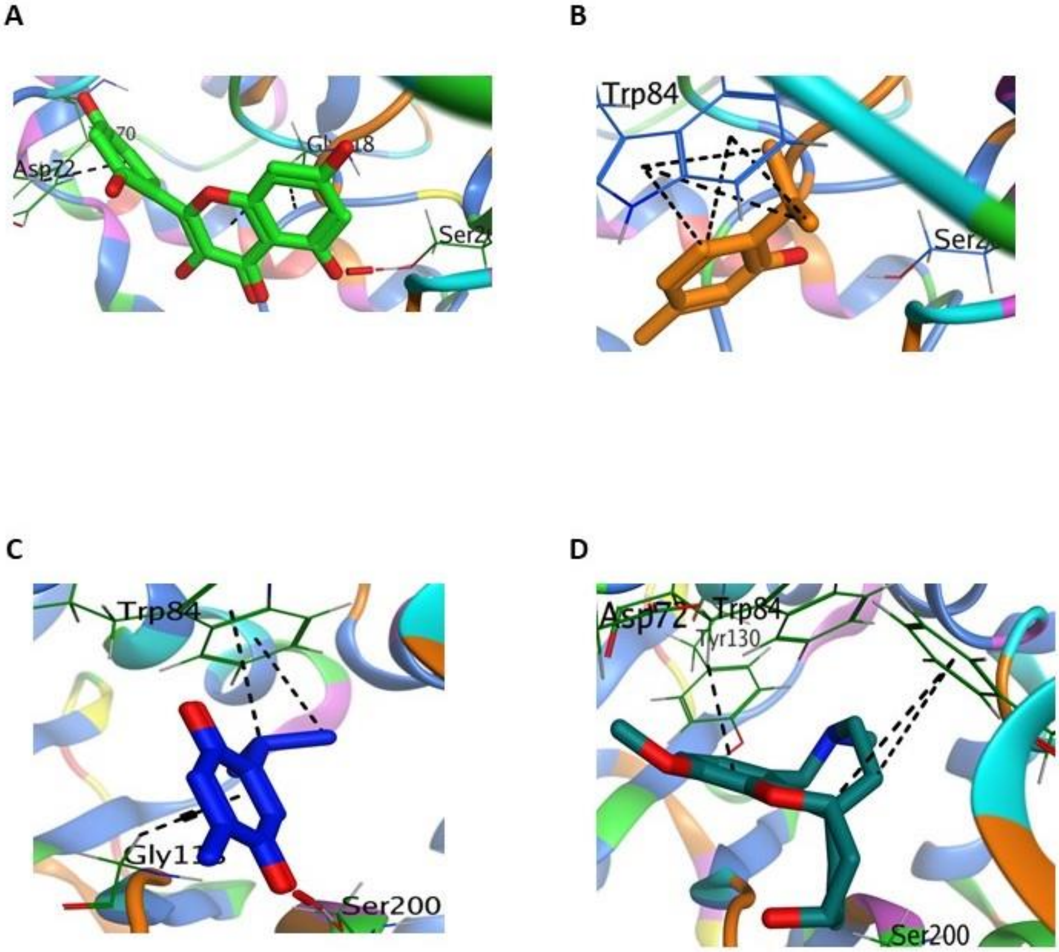

Figure S7. 3D docking poses of compounds (A) MOR (B) TML (C) TMQ (D) the ligand 3O0 into the 3O0 binding site in CDK5 PDB: 3O0G.

Supplementary data S7

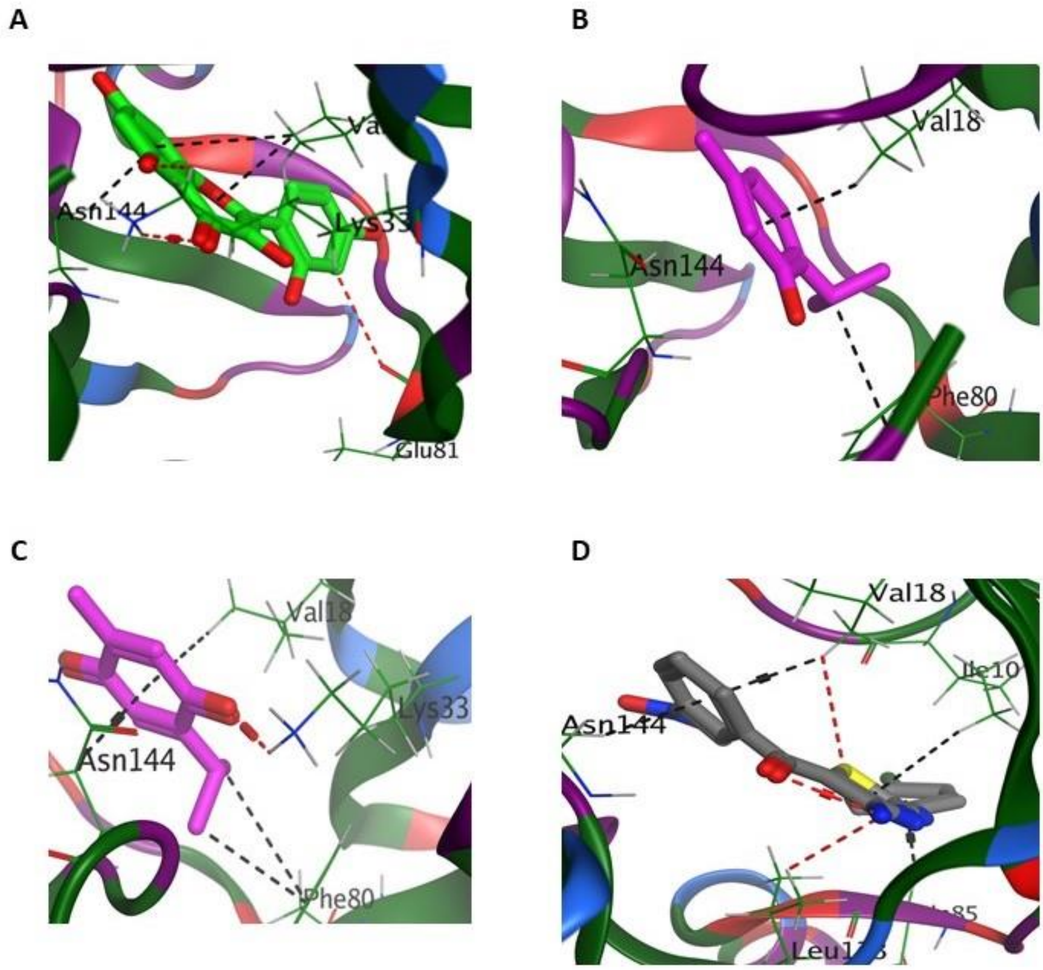

Figure S8. 3D docking poses of compounds (A) MOR (B) TML (C) TMQ (D) ligand 66F into the 66F binding site in BACE 1: 7D2V.

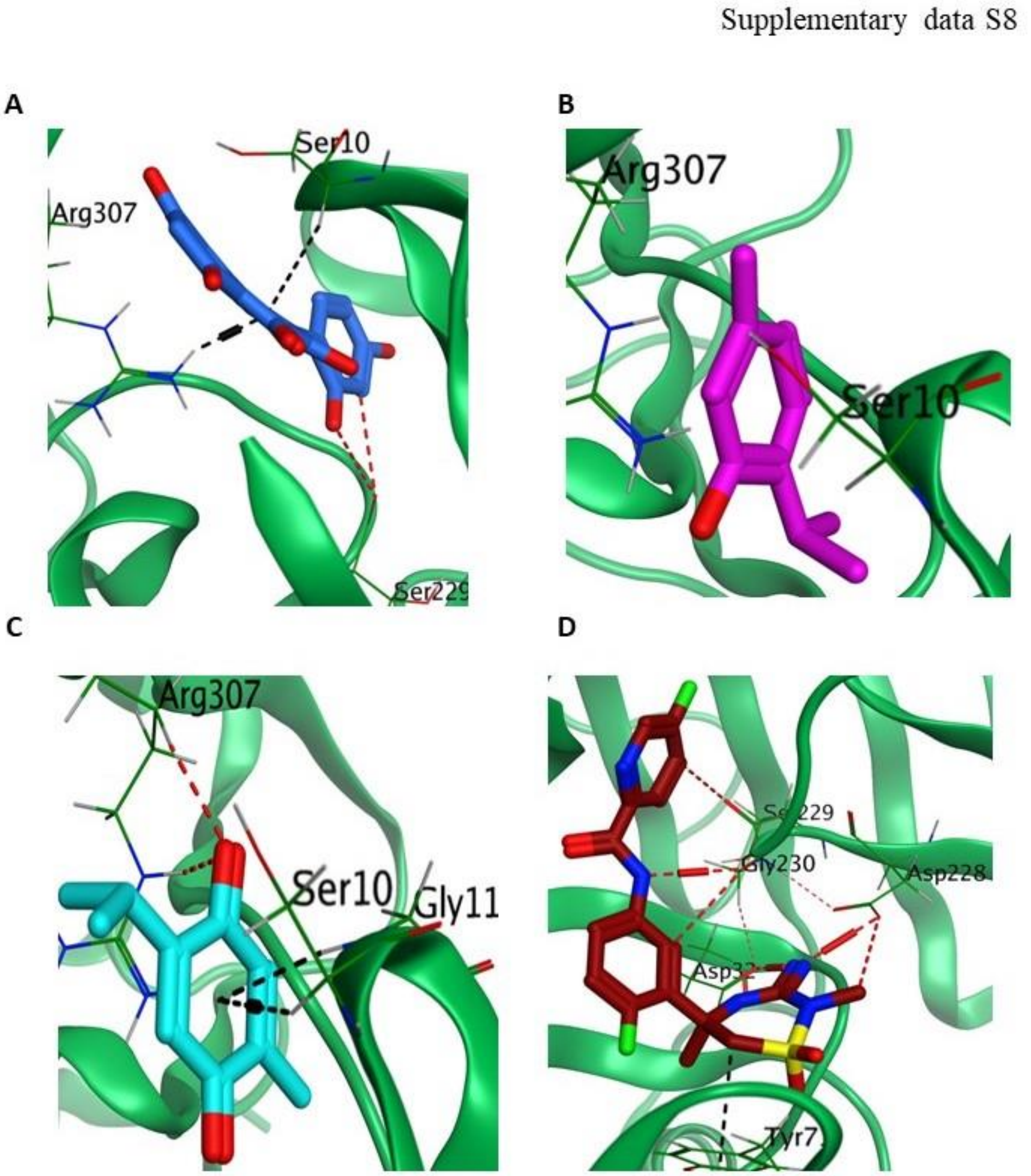

Figure S9. 3D docking of compounds (A) MOR (B) TML (C) TMQ (D) the ligand 65A into the 65A binding site in the glycogen synthase kinase 3 PDB: 5HLP.

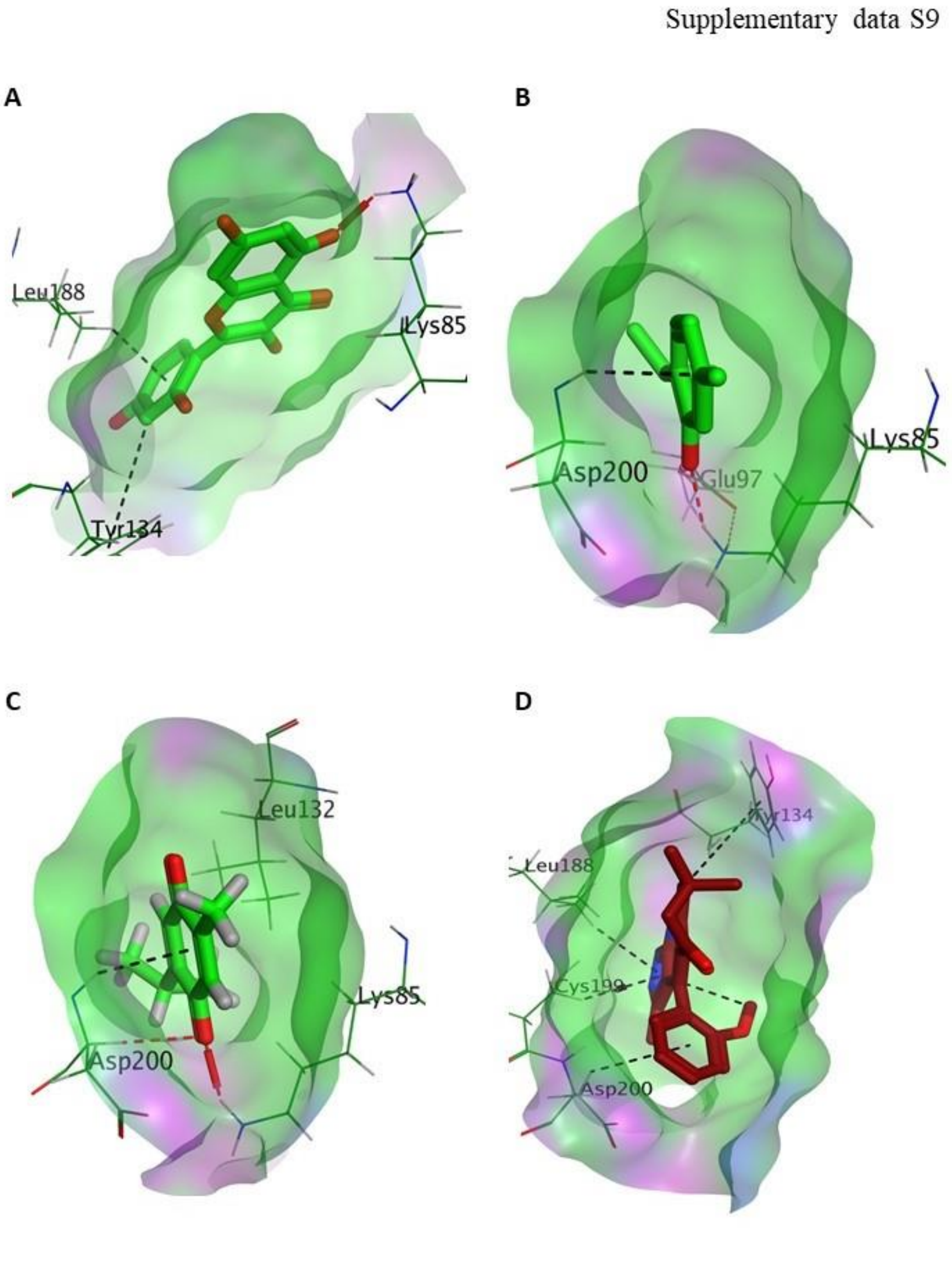

Figure S10. 3D docking of compounds (A) MOR (B) TML (C) TMQ (D) the ligand D8N into the D8N binding site in Keap1/5 complex (PDB ID 6FFM).

Supplementary data S10

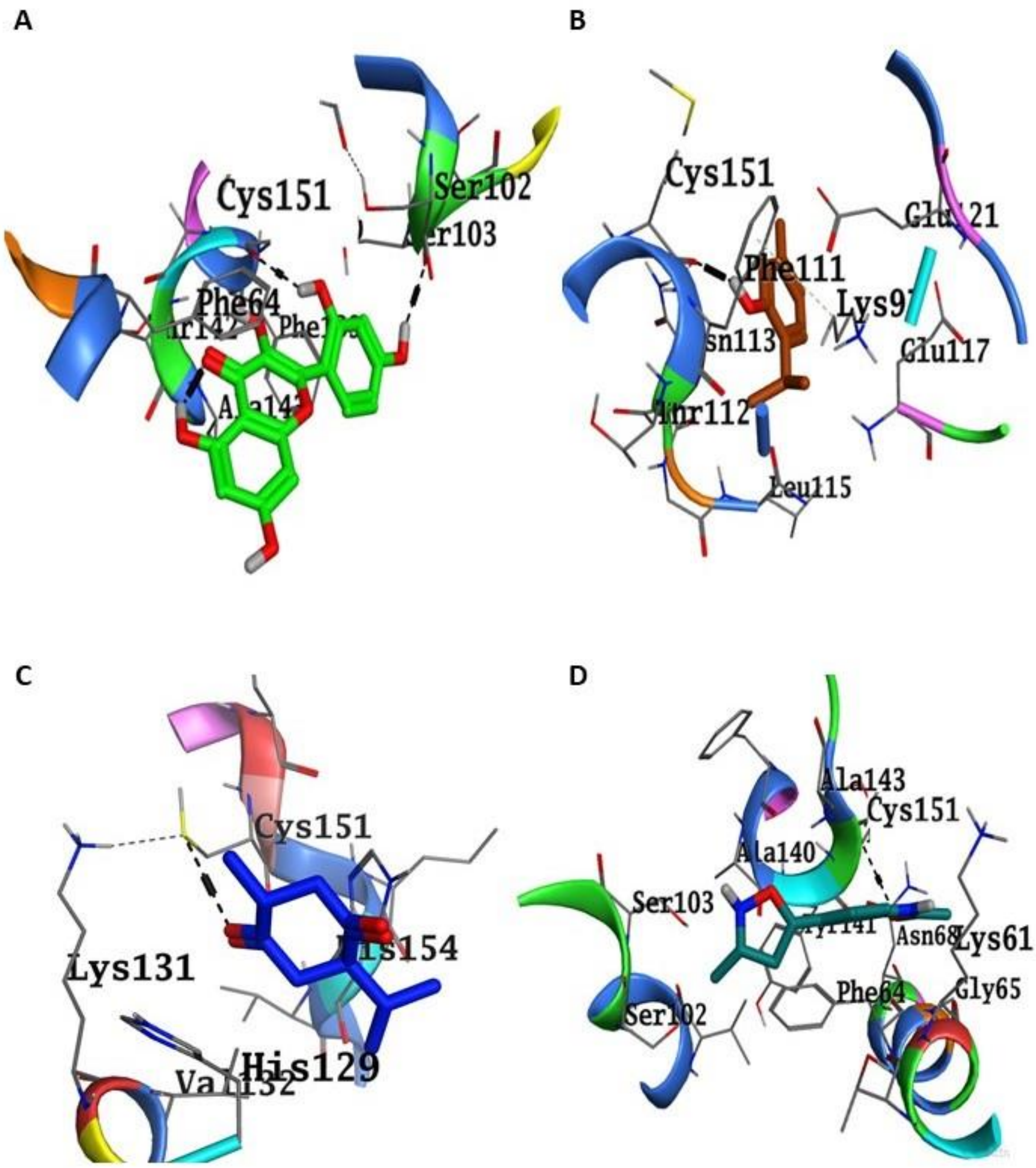

2) Tables

Table. S1. Receptor interaction of compounds A-C and galantamine into the galantamine binding site in the choline esterase PDB: 1DX6.

Compound	S	E_conf	rmsd_refine	Receptor
	dG Kcal/mole	Kcal/mole		Amino acid/Type of bonding/Distance (Å) / Binding Energy (Kcal/mole)
**1**	-6.4961	-71.3709	1.7066	TYR 70/ H-donor/3.03/-2.5<br>ASN 85/H-donor/3.32/-0.1<br>SER 200/H-acceptor/2.76/-3.2<br>PHE 331/H-acceptor/4.11/-0.1<br>HIS 440/Ionic/3.08/-4.0<br>ASP 72/pi-H/3.74/-0.2<br>GLY 118/pi-H /3.75/-0.4<br>GLY 118/pi-H/ 4.17/ -0.4
**2**	-4.8590	-21.0856	1.8786	GLU 199/H-donor/3.31/-0.1<br>GLU 199/H-donor/3.73/-0.1<br>TRP 84/H-pi/3.78/-0.4<br>TRP 84/H-pi/ 4.67/-0.3<br>TRP 84/H-pi/4.11/-0.1<br>TRP 84/H-pi/4.67/-0.2<br>TRP 84/H-pi /4.81/-0.2<br>TRP 84/H-pi/4.21/-0.3<br>GLY 118/pi-H/4.77/-0.1
**3**	-5.3373	1.4910	1.6847	TYR 130/H-donor/3.64/-0.1<br>GLU 199/H-donor/3.56/-0.1<br>GLU 199/H-donor/3.55/-0.1<br>HIS 440/H-donor/3.68/-0.1<br>SER 200/H-acceptor/2.83/-2.0<br>TRP 84/H-pi/3.77/-0.1<br>TRP 84/H-pi/4.27/-0.2

				TRP 84/H-pi/3.87/-0.3<br>GLY 118/pi-H/3.60/-0.8
**Galantamine**	-7.1875	21.0709	1.4719	TRP 84/H-donor/3.39/-0.1<br>GLU 199/Ionic/3.91/-0.7<br>PHE 330/H-pi/4.75/-0.2<br>PHE 330/H-pi/4.62/-0.2<br>TRP 84/pi-H/3.86/-0.2

Table. S2. Receptor interaction of compounds 1-3 and the ligand 3O0 into the 3O0 binding site in the Cdk5 PDB: 3O0G.

**Compound**	**S**	**E_conf**	**rmsd_refine**	**Receptor**
	**dG Kcal/mole**	**Kcal/mole**		**Amino acid/Type of bonding/Distance** (Å) **/ Binding Energy (Kcal/mole)**
**1**	-6.5722	-77.1360	1.5296	GLN 130/4.00/-0.1<br>GLU 81/H-donor/3.65/-0.2<br>LYS 33/H-acceptor/3.16/-0.2<br>VAL 18/H-acceptor/3.90/-0.1<br>LYS 33/H-acceptor/3.61/-1.0<br>LYS 33/Ionic/2.91/-5.1<br>VAL 18/pi-H/3.96/-0.1<br>VAL 18/pi-H/4.08/-0.5<br>VAL 18/pi-H/4.75/-0.2<br>ASN 144/pi-H/4.34/-0.1<br>ASN 144/pi-H/3.49/-0.3
**2**	-5.4249	-20.2428	0.8701	ALA 31/H-donor/4.12/-0.1<br>GLY 11/H-donor/3.47/-0.1<br>GLY 11/H-donor/3.62/-0.1<br>PHE 80/H-pi/3.68/-0.5<br>VAL 18/pi-H/3.63/-0.5<br>VAL 18/pi-H/4.27/-0.1<br>ASN 144/pi-H/3.97/-0.1
**3**	-5.3995	1.5897	1.6084	ASN 131/H-donor/3.65/-0.1<br>VAL 18/H-acceptor/3.51/-0.1<br>LYS 33/H-acceptor/3.08/-1.5<br>PHE 80/H-pi/4.36/-0.3<br>PHE 80/H-pi/3.66/-0.5<br>VAL 18/pi-H/4.33/-0.5<br>ASN 144/pi-H/3.63/-0.7

**Ligand**	-7.7325	-28.5348	1.8423	CYS 83/H-donor/3.14/-2.0<br>CYS 83/H-donor/3.14/-2.0<br>GLU 81/H-donor/3.49/-0.4<br>GLU 81/H-donor/3.49/-0.4<br>VAL 18/H-acceptor/4.32/-0.2<br>VAL 18/H-acceptor/4.36/-0.2<br>CYS 83/H-acceptor/3.86/-0.3<br>CYS 83/H-acceptor/3.86/-0.3<br>LEU 133/H-acceptor/3.58/-0.2<br>LEU 133/H-acceptor/3.51/-0.2<br>ILE 10/pi-H/4.46/-0.4<br>VAL 18/pi-H/4.25/-0.7<br>GLN 85/pi-H/3.81/-1.0<br>ASN 144/pi-H/3.99/-0.2<br>ILE 10/pi-H/4.56/-0.5<br>VAL 18/pi-H/4.22/-0.7<br>GLN 85/pi-H/3.81/-1.0<br>ASN 144/pi-H/3.95/-0.3

Table. S3. Receptor interaction of compounds 1-3 and the ligand 66F into the 66F binding site in the BACE 1, PDB: 7D2V.

**Compound**	**S**	**E_conf**	**rmsd_refine**	**Receptor**
	**dG Kcal/mole**	**Kcal/mole**		**Amino acid/Type of bonding/Distance** (Å) **/ Binding Energy (Kcal/mole)**
**1**	-5.6644	-74.9125	1.3282	GLY 11/H-donor/3.13/-0.1<br>GLY 11/H-donor/3.13/-0.1<br>SER 229/H-donor/3.51/-0.4<br>SER 229/H-donor/3.51/-0.4<br>SER 229/H-donor/3.32/-0.4<br>SER 229/H-donor/3.32/-0.4<br>ARG 307/Ionic/3.80/-1.0<br>ARG 307/Ionic/3.80/-1.0<br>SER 10/pi-H/4.62/-0.3<br>ARG 307/pi-cation/3.21/-2.1<br>SER 10 /pi-H/4.62/-0.3<br>ARG 307/pi-cation/3.21/-2.1
**2**	-5.39809465	1.68075621	-15.7678709	THR 232/H-donor/3.62/-0.1<br>THR 232/H-donor/3.62/-0.1<br>SER 10/pi-H/4.09/-0.1<br>ARG 307/ pi-H/ 4.97/-0.1<br>SER 10/pi-H/4.09/-0.1<br>ARG 307/pi-H/ 4.97/-0.1
**3**	-5.0369	3.0587	1.6280	SER 10/H-donor/3.37/-0.1<br>SER 10/H-donor/3.37/-0.1<br>GLU 339/H-donor/4.03/-0.1<br>GLU 339/H-donor/4.03/-0.1<br>VAL 170/H-acceptor/3.54/-0.1<br>VAL 170/H-acceptor/3.63/-0.1

				VAL 170/H-acceptor/3.54/-0.1<br>VAL 170/H-acceptor/3.63/-0.1<br>ARG 307/H-acceptor/3.44/-0.2<br>ARG 307/H-acceptor/3.22/ -0.2<br>ARG 307/H-acceptor/3.44/-0.2<br>ARG 307/H-acceptor/3.22/-0.2<br>SER 10/pi-H/3.99/-0.9<br>GLY 11/pi-H/3.44/-0.2<br>SER 10/pi-H/3.99/ -0.9<br>GLY 11/ pi-H/3.44/-0.2
**Ligand 66F**	-8.0110	-345.3253	1.57711	ASP 228/H-donor/3.47/-0.2<br>ASP 228/H-donor/3.47/ -0.2<br>ASP 32/H-donor/2.92/-7.5<br>ASP 228/H-donor/2.94/-9.1<br>ASP 32/H-donor/2.92/-7.5<br>ASP 228/H-donor/2.94/ -9.1<br>ASP 32/H-donor/2.93/-2.9<br>ASP 32/H-donor/2.93/-2.9<br>GLY 230/H-donor/3.39/-0.2<br>GLY 230/H-donor/3.39/-0.2<br>GLY 230/H-donor/3.07/-3.0<br>GLY 230/H-donor/3.07/-3.0<br>SER 229/H-donor/3.37/-0.3<br>SER 229/H-donor/3.37/-0.3<br>GLY 11/H-donor/3.21/-0.1<br>GLY 11/H-donor/3.21/-0.1

				LEU 30/H-acceptor/3.66/-0.1<br>TRP 115/H-acceptor/3.59/-0.1<br>LEU 30/H-acceptor/3.66/-0.1<br>TRP 115/H-acceptor/3.59/-0.1<br>PHE 108/H-acceptor/3.32/-0.1<br>PHE 108/H-acceptor/3.32/-0.1<br>ASP 228/Ionic/ 4.21/-0.2<br>ASP 228/Ionic / 4.21/-0.2<br>ASP 32/Ionic/2.92/-5.0<br>ASP 32/ Ionic /3.64/-1.4<br>ASP 228/Ionic/3.04/-4.2<br>ASP 228/Ionic/2.94/-4.9<br>ASP 32/Ionic/2.92/-5.0<br>ASP 32 / Ionic/3.64/-1.4<br>ASP 228/Ionic/ 3.04/-4.2<br>ASP 228/Ionic/2.94/-4.9<br>ASP 32 /Ionic/3.63/-1.4<br>ASP 32/Ionic/2.93/-4.9<br>ASP 32 / Ionic/3.63/-1.4<br>ASP 32/ Ionic/2.93/-4.9<br>TYR 71/H-pi/3.67/-0.5<br>TYR 71/H-pi /3.67/ -0.5

Table. S4. Receptor interaction of compounds 1-3 and the ligand 65A into the 65A binding site in the glycogen synthase kinase 3 PDB: 5HLP.

**Compound**	**S**	**E_conf**	**rmsd_refine**	**Receptor**
	**dG**<br>**Kcal/mole**	**Kcal/mole**		**Amino acid/Type of bonding/Distance** (Å) **/ Binding Energy (Kcal/mole)**
**1**	-5.4860	-78.8546	1.89496088	VAL 135/H-donor/3.31/-0.1<br>LYS 85/H-acceptor/3.26/-0.1<br>LYS 85/H-acceptor/2.85/-12.7<br>VAL 70/H-acceptor/3.55/-0.1<br>LYS 85/Ionic/2.85/-5.6<br>TYR 134/H-pi/4.89/-0.2<br>LYS 85/pi-H/4.88/-0.1<br>LEU 188/pi-H/3.55/-0.2<br>CYS 199/pi-H/4.42/-0.1<br>ASP 200/pi-H/3.57/-0.1
**2**	-5.1836	-18.9100	1.4032954	GLU 97/H-donor/3.16/-2.2<br>LYS 85/H-acceptor/3.05/-0.4<br>LEU 132/pi-H/3.87/-0.1<br>CYS 199/pi-H/4.31/-0.1<br>ASP 200/pi-H/3.72/-0.4
**3**	-5.3032	2.7724	0.8087	GLU 97/H-donor/3.24/-0.1<br>MET 101/H-donor/3.69/-0.1<br>CYS 199/H-donor/4.36/-0.1<br>VAL 110/H-acceptor/3.97/-0.1<br>LEU 188/H-acceptor/3.97/-0.1<br>LYS 85/H-acceptor/3.34/-0.1<br>LYS 85/H-acceptor/3.07/-7.6<br>ASP 200/H-acceptor/3.46/-0.2<br>LEU 132/pi-H/3.87/-0.1

				CYS 199/pi-H/4.25/-0.1<br>ASP 200/pi-H/3.76/-0.2
**65A**	-<br>6.56960344		1.8019	CYS 199/H-donor/4.32/-0.1<br>VAL 135/H-donor/3.54/-0.1<br>PRO 136/H-donor/3.35/-0.1<br>LEU 188/H-acceptor/3.82/-0.1<br>TYR 134/H-pi/4.97/-0.3<br>LEU 132/pi-H/4.21/-0.1<br>LEU 188/pi-H/4.29/-0.3<br>LEU 188/pi-H/4.53/-0.1<br>LEU 188/pi-H/4.06/-0.1<br>CYS 199/pi-H/3.94/-0.9<br>ASP 200/pi-H/4.27/-0.3
